# Molecular understanding of the morphology and properties of lignin nanoparticles: unravelling the potential for tailored applications†

**DOI:** 10.1039/d3gc00703k

**Published:** 2023-05-12

**Authors:** Ievgen V. Pylypchuk, Maria Karlsson, Pär A. Lindén, Mikael E. Lindström, Thomas Elder, Olena Sevastyanova, Martin Lawoko

**Affiliations:** a Division of Wood Chemistry and Pulp Technology, Department of Fiber and Polymer Technology, School of Chemistry, Biotechnology and Health, KTH Royal Institute of Technology Teknikringen 56-58 100 44 Stockholm Sweden olena@kth.se lawoko@kth.se +468 7908066 +46 767 762 735 +46 8 7908047 +46 73 4607647; b Department of Materials and Environmental Chemistry, Stockholm University Svante Arrhenius väg 16C 10691 Stockholm Sweden; c Wallenberg Wood Science Center, Department of Fiber and Polymer Technology, School of Chemistry, Biotechnology and Health, KTH Royal Institute of Technology Teknikringen 56-58 100 44 Stockholm Sweden; d USDA-Forest Service, Southern Research Station 521 Devall Drive Auburn AL 36849 USA

## Abstract

Studies have shown that the size of LNP depends on the molecular weight (*M*_w_) of lignin. There is however need for deeper understanding on the role of molecular structure on LNP formation and its properties, in order to build a solid foundation on structure–property relationships. In this study, we show, for similar *M*_w_ lignins, that the size and morphology of LNPs depends on the molecular structure of the lignin macromolecule. More specifically, the molecular structure determined the molecular conformations, which in turn affects the inter-molecular assembly to yield size- and morphological-differences between LNPs. This was supported by density functional theory (DFT) modelling of representative structural motifs of three lignins sourced from Kraft and Organosolv processes. The obtained conformational differences are clearly explained by intra-molecular sandwich and/or T-shaped π–π stacking, the stacking type determined by the precise lignin structure. Moreover, the experimentally identified structures were detected in the superficial layer of LNPs in aqueous solution, confirming the theoretically predicted self-assembly patterns. The present work demonstrates that LNP properties can be molecularly tailored, consequently creating an avenue for tailored applications.

## Abstract 2

Studies have shown that the size of LNP depends on the molecular weight (*M*_w_) of lignin.

## Graphical abstract

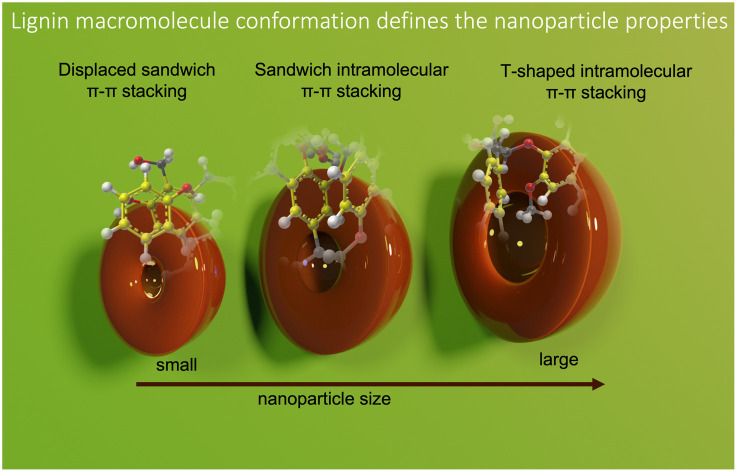

## Introduction

Around 15–30% of the lignocellulosic biomass consists of lignin, which is the most abundant aromatic polymer in nature.^1^ Historically, lignin is a by-product of the chemical pulp production process, which is optimized towards high-quality fibers. In the context of transitioning towards a bioeconomy however, the interest in using lignin as a source of aromatics and replacement for fossil sources has grown and is the subject of several research efforts. In effect, different strategies have been, and will continue to be explored, leading to the further development of the concepts of the biorefinery.^2,3^

Lignin has been proposed for many applications, including highly reinforced biodegradable composites,^4^ tough hydrogel adhesives,^5^*etc*. Lignin nanoparticles (LNPs) in particular have been proposed for many applications, such as food additives,^6^ packaging,^7^ pharmaceutical and cosmetic industries.^8^ Manufacturing costs for LNPs are estimated to be *ca.* 1000 USD/t,^9^ and if they are applied as UV-protecting agents^10^ or Pickering emulsions^11,12^ stabilizers and for biocatalysis.^13^The potential profit from LNP can reach *ca.* 1000 USD per t if the revenue is 2000 USD per ton, according to Balakshin *et al*.^14^ The applications of LNPs were reviewed by Osterberg,^15^ Sipponen^16^ and others.^17,18^ Recent achievements in preparation of LNPs at high consistency have been reported by Pylypchuk.^19^

The structure of lignin is altered depending on the pulping process and the method of lignin extraction. The kraft process is the most common chemical pulping process.^20^ The lignin, ending up in the black liquor, can be recovered through various processes, including LignoBoost or LignoForce process.^21^ However, the lignin retrieved from the existing technical processes still suffer from heterogeneity and severe structural alterations compared to native wood lignin as a natural consequence of the pulping process.^22^ Another way to produce pulp is by the organosolv process, developed in the 1970s.^23^ The principle is to use an aqueous organic extraction solvent, with or without the addition of a catalyst, to remove the lignin from the fibers.^3^

The processes for lignin extraction can be further developed to target the high quality of several parts of the biomass and not only the fiber, in a biorefinery concept, where several parts of the biomass can serve as raw materials for different applications. In this context, physical and chemical protection strategies have been adopted to preserve the original lignin structure. These include flow-through processes for physical protection, or reactive-site end-capping additives that prevent repolymerizations and condensation reactions during the extraction process.^24–26^

Although LNPs have been studied, a detailed account on how the molecular structure of lignin may affect the assembly mechanisms as well as LNP properties is still lacking. This information is required to gauge the potential to tailor the molecular properties of LNPs, which outlines the content of the present study. Accordingly, LNPs sourced from three spruce lignins differing in structure and, derived from three different extraction processes where studied. The processes were kraft, classical ethanol organosolv and an novel ethanol organosolv based on physical and chemical protected strategies.^27^

All three lignins were further refined by ethanol fractionation at ambient temperature in order to obtain more homogeneous fractions and improve the characterization of molecular properties of the lignin that would consequently enable a better understanding of the influence of the lignin chemical structure on the LNPs self-assembly, size, surface morphology and surface functionality. Different NMR techniques were used for the characterization of the lignin and the surface of the LNPs. The analyses provided mechanistic insights into the process of formation of lignin nanoparticles and the chemical structure of their surface in an aqueous solution. These insights, with strong support from DFT modelling of representative structural motifs, unravel the important role of molecular structure in LNP properties.

## Experimental

### Materials and chemicals

Wood chips were obtained from Norway spruce (*Picea abies*). Kraft lignin from Norway spruce (*Picea abies*) was obtained from processed black liquor in the LignoBoost process.

All water used in the experiments was Milli-Q water (Millipore, Q-POD, Millipak 0.22 μm filter). Ethanol (absolute) and Tetrahydrofuran (HPLC grade) were purchased from VWR chemicals. Sulfuric acid (>95%, analytical grade) and acetic anhydride (99.7%, analytical grade) from Fischer chemicals. Pyridine (anhydrous, 99.8%), *endo-N*-hydroxy-5-norbornene-2,3-dicarboximide (*e*HNDI; 97%), chromium(iii) acetylacetonate (Cr(acac_3_); 99.99%), 2-chloro-4,4,5,5-tetramethyl-1,3,2-dioxaphospholane (Cl-TMDP; 95%), [D_6_] DMSO (99.9 at% D), *N,N*-dimethylformamide (anhydrous, 99.8%), CDCl_3_ (≥99.8 at% D) were purchased from Sigma–Aldrich. Acetone (VWR chemicals, lot #19F064007); 0.45 μm membrane filters (Fisherbrand, PTFE, 0.45 μm) were used.

## Methods

### Preparation of wood sample

The spruce wood chips were ocularly examined and debarked to collect only bright wood without any visible defect. The selected wood chips were Wiley milled to a size of 40 mesh (Wiley mini-mill, 3383L70, Thomas Scientific).

### Extraction and preparation of lignin

The extraction of the OS (organosolv) protected, and the OS reference lignin samples were performed using an ASE 350 Accelerated Solvent Extractor (Dionex, Sunnyvale, CA, USA). The 66 mL dionium zirconium extraction cell was used for the wood sample extraction. To collect the extract, 250 ml bottles were used.

The extraction method was performed as has been presented elsewhere.^27^ Briefly, in the 66 ml zirconium cell, 9.281 g (oven-dried basis) of the Wiley milled wood was placed into the cell together with the glass fiber filters. In the first extraction step, a 2 h HW (hot water) extraction was performed using Milli-Q water as extraction solvent, followed by the lignin extraction. The HW extraction was performed at 160 °C using a fixed volume of 70 mL and a purge time of 90 s.

The HW extraction was followed by the lignin extraction, where a physical protection design is used to protect the lignin structure without the need of chemical additives. The extraction design is based on a cyclic process, where only a part of the liquid is replaced every 5 minutes with a new solvent, thereby preventing extensive depolymerization. An additional effect is a less saturated extract which prevents lignin repolymerization. The OS protected lignin extraction was performed using a solvent system of aqueous ethanol (30 : 70 v/v) and using 1.5 wt% of H_2_SO_4_ as a catalyst. The extraction was performed in a total of 15 cycles at 5 min each, using 9 + 6 static cycles at 160 °C, rinse volume of 100% and 60% respectively and a purge time of 90 s. Between the 9 and 10 cycles, the extraction solvent in the cell was emptied and the new solvent was delivered and heated for 8 minutes since the instrumental limit of cycles for the instrument is 9.

The OS reference lignin extraction was performed using the same solvent system, *i.e.*, aqueous ethanol (30 : 70 v/v) and 1.5 wt% of H_2_SO_4_ using an extraction time of 2 h at 160 °C, and 1 static cycle and a purge time of 90 s.

All the extractions were performed at a pressure of 1500–1600 psi.

The ethanol in the lignin extracts was evaporated under reduced pressure. To prevent further reactions due to increased acidity during evaporation, water was added to each extract before and under evaporation and the pH was monitored. The precipitated lignin was filtered under vacuum using a glass fiber filter and rinsed with water to remove carbohydrates. The lignin was collected from the filter and dried in a vacuum oven without heating for 40 h. The HW extracts were directly lyophilized.

The dry content of the Wiley milled wood and the vacuum-dried lignin samples was determined by drying an aliquot in an oven overnight at 105 °C. The dry weight after treatment in the vacuum oven was determined to be 0.95%. The dry content for the extracted lignin samples was determined to be 99%.

### Fractionation of lignin

An aliquot of the lignin sample was fractionated using ethanol (absolute), with a lignin/ethanol ratio of (1 : 40 w/w). The solution was placed in a closed bottle and stirred using a magnet for 2 h. The solution was directly filtered under vacuum. A small amount of ethanol was used to rinse the filter. The EtOH in the soluble lignin fraction in the filtrate was evaporated under reduced pressure. The fractions were further dried in a vacuum oven without heating to ensure that all ethanol was evaporated. Lignin samples were named as OS p., OS ref. and kraft. Indexes 1,2,3 corresponds to soluble, initial and insoluble fractions respectively.

### Lignin nanoparticles preparation

The lignin from each lignin fraction was dissolved in an acetone: water mixture (4 : 1, v/v) to give a solution with a concentration of 5 mg ml^−1^ lignin. Samples were filtered through a 0.45 μm membrane filter to remove undissolved material and possible aggregates. After filtration and drying for 24 h at 60 °C the filters were weighed. Filtered solutions with a concentration of 1 mg ml^−1^ lignin were used for LNPs preparation (the lignin concentration in filtered solution was calculated taking into account the weight of the adsorbed lignin materials on the filter). Under moderate stirring, 4 ml of deionized water was then added dropwise to 1 ml of lignin solution in acetone : water (4 : 1, v/v), and the solution was stirred until all acetone had evaporated.

### DFT modelling

The lignin oligomers examined using computational chemical methods contain 5–6 aromatic rings and a range of interunit linkages. Given the flexibility and number of rotatable bonds, an initial 500 step Monte Carlo conformational search was performed with optimization using the MMFF force-field. The unique conformations identified were further optimized with the PM6 semi-empirical method. Both of these steps were done as implemented in Spartan '20.^28^ The 10 lowest energy conformations from the PM6 calculations were further refined with optimization using the M06-2X density functional method, the GD3 empirical dispersion correction and the 6-31+G(d,p) basis set as implemented in Gaussian16.^29^ The lowest energy gas phase conformation was used as the starting geometry for optimization in water and acetone using the SMD model. Measurements of inter-ring distances were done with Mercury 2022.1.0^30^ and molecular volumes were calculated with Spartan'20.^28^

### Size exclusion chromatography (SEC)

For the estimation of the molecular weight distribution and dispersity index, size-exclusion chromatography was performed using a Waters instrument (Waters Milford, MA, USA) consisting of a 2707 autosampler, a 515 HPLC pump and a 2998 photodiode array detector (PAD) operated at 254 and 280 nm.

All lignin samples were acetylated prior to analysis to overcome eventual solubility problems in THF. The acetylation of the lignin samples was performed as follows: 2.5 mg of lignin was dissolved in 100 μL of acetic anhydride and 100 μL of pyridine (1 : 1, v/v). The sample mixture was placed into a thermomixer operated without heating at 400 rpm overnight. Ethanol (absolute) was added dropwise before and during evaporation of the solvents using a stream of nitrogen. Finally, the dried residue was dissolved in 1 mL THF. All acetylated lignin samples were filtered using a 0.2 μm NYL syringe filter.

HPLC-grade THF was used as the mobile phase using a flow rate of 0.3 mL min^−1^. The column system consisted of a Waters Styragel Guard column (4.6 × 300 mm) connected in series with Waters Ultrastyragel HR4, HR2 and HR0.5 (4.6 × 300 mm) solvent efficient columns, operated at a column temperature of 35 °C. The injection volume was 20 μL.

Calibration was performed at 254 nm using polystyrene standards with nominal molecular weights ranging from 162 to 176 000 Da (specifically; 176 000, 116 000, 46 400, 18 000, 9600, 6540, 2920, 890, 578, 474, 370, 266 and 162 Da). Data analysis and quantification were performed using the Waters Empower 3 build 3471 software.

### Nuclear magnetic resonance (NMR) spectroscopy of lignin fractions

Liquid state NMR spectroscopy of the lignin fractions was acquired on a Bruker NMR spectrometer Avance III HD 400 MHz instrument (Bruker Corporation, Billerica, MA, USA) equipped with a *Z*-gradient and a 5 mm BBFO broadband smart probe (Bruker Corporation, Billerica, MA, USA). For each fraction, 80 mg of lignin was dissolved in 600 μl DMSO-d_6_.

The HSQC experiments were performed using the “hsqcetgpsi” pulse program using 86 scans (and 16 additional dummy scans) over 1024 × 256 increments, a relaxation delay of 1.5 s, an acquisition time of 0.087 s and a spectral window of: 166.7 ppm on F1 and 14.7 ppm on F2. The experiments were performed at a temperature of 298 K.

The data was processed in MestReNova (version 9.0.0, Mestrelab Research) using 1024 × 1024 data points using a 90°-shifted square sine-bell apodization window. The data was Fourier transformed followed by phase correction and baseline correction which was applied in both dimensions by means of a Bernstein polynomial fit of order 3.

The semi-quantification of lignin interunit linkages was performed using the C2-signal region on the aromatic ring as an internal standard.^31^

All NMR spectra were integrated using the same shifts for comparable results (Table S1†).

The ^31^P NMR sample preparation was based on a previously reported method.^32,33^ An amount of 30 mg lignin was dissolved in equal volumes of *N*-dimethylformamide and pyridine (100 μl each). After dissolution, 50 μl internal standard solution (60 mg ml^−1^ eHNDI and 5 mg ml^−1^ Cr(AcAc_3_) relaxing agent dissolved in pyridine) was added. To the well stirred solution, approximately 100 μl of Cl-TMDP phosphorylating agent was added, followed by dropwise addition of 450 μl CDCl_3_.

The ^31^P NMR was performed using 256 scans, a relaxation delay of 5 s and an acquisition time of 1.6777 s. The experiment was performed at a temperature of 298 K. The data was processed in MestReNova (version 9.0.0, Mestrelab Research) and Fourier transformed followed by phase correction and baseline correction by means of a Bernstein polynomial fit of order 3. Diagnostic signals and assigned shifts are presented in Tables S3–S5.† The assigned shifts are relative to the water reaction product of Cl-TMDP at 132.2 ppm.

The ^1^H NMR of the lignin fractions was performed by dissolving 80 mg of lignin in 600 μl DMSO-d_6_, using 80 scans, a relaxation delay of 5.5 s and an acquisition time of 0.5 s. The experiment was performed at a temperature of 298 K. The data was processed in MestReNova (version 9.0.0, Mestrelab Research) and Fourier transformed followed by phase correction.

### Dynamic light scattering (DLS)

The zeta potential, average size and size distribution of synthesized nanoparticles were determined for NPs “as prepared”, using a Zetasizer Nano ZS instrument (Malvern-Pananalytical, Malvern, UK) at 25 °C.

### Scanning electron microscopy (SEM)

The LNP solution was drop-cast on a silicon wafer for 30 min and sputter-coated with a 2 nm layer of Pt–Pd alloy. The LNPs were observed using an S-4800 scanning electron microscope (Hitachi, Japan) at accelerating voltage 5 kV and emission current 7.4 mA.

### Transmission electron microscopy (TEM)

TEM analysis was performed on a Hitachi HT7700 series instrument (Hitachi, Japan), at an accelerating voltage of 100.0 kV and an emission current of 8.0 μA. Samples were prepared in the following way: 5 μL of lignin NPs suspension (0.2 mg ml^−1^) was drop-cast on a 200-mesh copper grid (Micro to Nano, 22-1MFC20-50) and dried in air for 30 min.

### ^1^H NMR of LNPs

4.5 ml of lignin NPs suspension (*ca.* 0.22 mg ml^−1^ of lignin) was centrifuged using a tabletop centrifuge (Eppendorf, model “mini spin plus”) for 15 min at 14 200 rpm. The supernatant was removed by decantation and the precipitated NPs were redispersed in 0.6 ml of D_2_O. The procedure was repeated 3 times. The final concentration of lignin in the NPs suspension was *ca.* 1.5 mg ml^−1^.

NMR experiments were then conducted on the LNPs using a Bruker 400 DMX instrument (Bruker Corporation, Billerica, MA, USA) equipped with a 5 mm Bruker BBI probe (Bruker Corporation, Billerica, MA, USA). For each experiment, presaturation followed by an excitation sculpting through two consecutive 3–9–19 Watergate blocks was applied by means of a user-defined pulse sequence. The Watergate blocks used gradient ratios of 40 and 7, respectively with a binomial delay time (d19) of 200 μs. Optimal 90° pulse lengths were obtained for each sample by finding the pulse length corresponding to the 360° pulse for which the proton FID signal was lowest, and then dividing by four. The experiments were performed using 1024 scans with an acquisition time of 0.5 seconds and a relaxation delay time of 5.5 seconds. The resulting data was first processed in TopSpin (version 1.3, patch level 10, Bruker BioSpin) with 64k data points using a 0.3 Hz exponential multiplication apodisation window and after Fourier transformation and phase correction, the spectra were transferred to MestreNova (version 9.0.0, Mestrelab Research), where a baseline correction was applied.

## Results and discussion

**Fig. 1:**
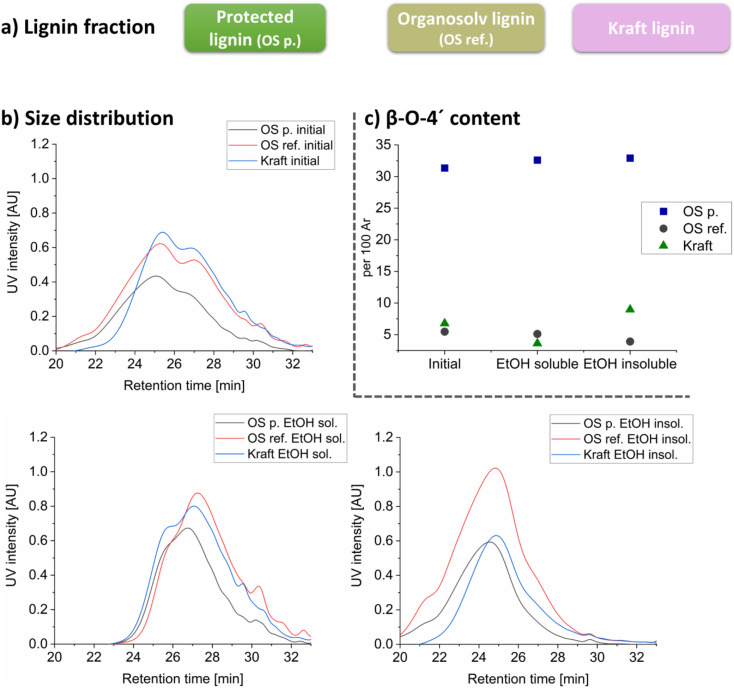
Comparison characterization data for lignins used in this work showing preservation of β-O-4′ in selected lignin. General overview of the experimental approach for lignin extraction (a), overlayed elution profile of the lignin fractions (b), and the trend of β-O-4′ content for the different lignin fractions, quantified by 2D HSQC, Ar = aromatic rings (c).

In this study, the differences in the chemical and physical properties of spruce lignin from three different extraction processes were first investigated. Subsequently, LNPs were synthesized from them. The main goal was to gain deeper fundamental insights into the mechanisms of LNP formation and unravel the role of molecular structure in the structural hierarchy of LNP. The studied lignins were the kraft lignin (LignoBoost), recovered from the kraft process, lignin from a classical ethanol-based organosolv process and finally, an ethanol-based organosolv performed in a cyclic extraction mode, according to a recently published method.^23^ The scheme for the cyclic extraction is shown Fig. S30 and S31.† The initial lignins are summarized in Fig. 1a. Noteworthy is the naming “Protected lignin” (Fig. 1a) is due to that the extracted lignin structure has an abundance of native linkages due to physical protection achieved by the cyclic extraction approach. All the initial lignins were further fractionated with absolute ethanol at ambient temperature to obtain “soluble” and “insoluble fractions”.

The molecular weight distribution of the lignin fractions investigated by SEC is shown in Fig. 1b and the molecular weight and dispersity indices are reported in Table S6 and Fig. S10,† respectively. All three initial (crude) lignin, *i.e.*, the OS protected, OS reference and kraft lignin show similar elution profiles (Fig. 1b) but with differences in peak broadness. The elution profiles of the ethanol-soluble fractions are similar but different from those of the ethanol-insoluble fractions, Fig. 1b. The lower molecular weight fractions were found in the ethanol soluble part while the higher molecular weight fractions were present in the insoluble fraction (Table S6†). The dispersity indexes were also reduced by refining for all the three lignins (Table S6†), with all ethanol soluble fractions having impressive value <2.

### Advanced 1D and 2D NMR studies reveal significant differences in molecular lignin structure

**Fig. 2:**
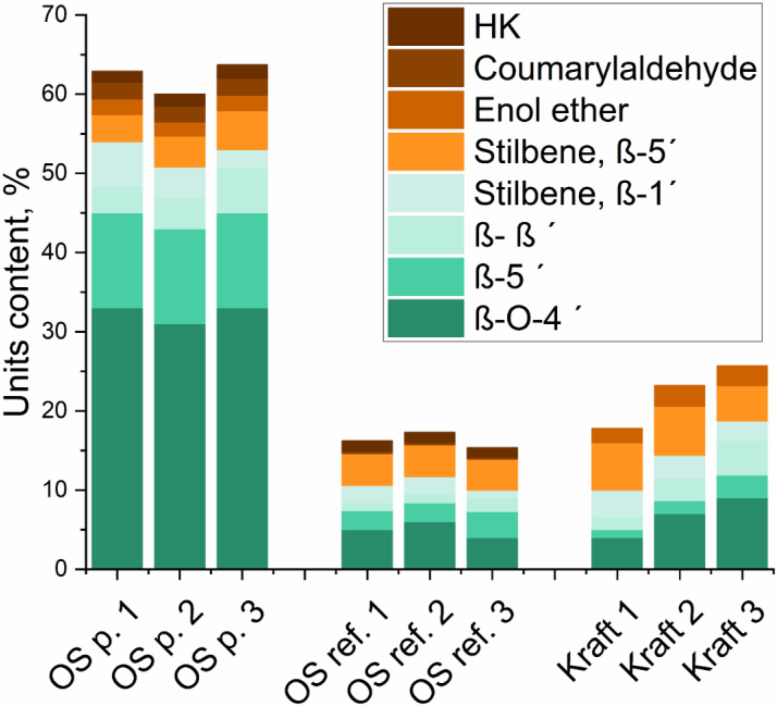
Quantification of inter-units in the three lignins, Fig. S38,† using the C2-aromatic region as an internal standard [per 100 Ar]. Indexes 1, 2, 3 corresponds to soluble, initial and insoluble fractions respectively.

All lignin fractions were characterized by ^31^P NMR and 2D HSQC NMR. In general, the aryl ether linkage (β-O-4′) content is significantly higher for the OS protected lignin compared to the other lignins from the extraction processes, demonstrating the mildness of the extraction due to the cyclic design of the process. The β-O-4′ content in the different crude lignins and their refined ethanol fractions are shown in Fig. 1c. The β-O-4′ content does not differ significantly between the refined fractions for each of the lignin types. In general, only the OS protected extracted lignins had significant presence of native linkages. This is due to the physical protection nature of the extraction method used.^23^ The HSQC analysis of the soluble fractions along with their assigned chemical structures are presented in Fig. 2. All HSQC spectra are also presented in Fig. S1–S9, and assignments in Table S1.†

The types and content of inter-units linkages in the different lignins are shown in Fig. 2 and in Table S2.†

The main inter-unit linkages in native lignin are the β-O-4′ linkage. In acid-catalyzed organosolv extraction, one depolymerization reaction is the acidolysis and the formation Hibbert's ketone (HK),^34,35^ which was detected in the organosolv extracted lignins, albeit at low concentrations, but not in the kraft samples. Other reactions of β-O-4′ bonds in organosolv extractions include homolytic cleavages and subsequent couplings yielding new carbon–carbon bonds.^27^ The products of such reactions would typically be found in the OS reference sample. In addition, the OS reference lignin differs from the OS protected lignin in the levels of condensations on the side chain. These condensations have been shown to occur mainly between position C6 of aromatic ring in guaiacyl nuclei and Cα of lignin, and here identified in the spectrum of the OS reference lignin, Fig. S4,† with the signals marked by dotted red rectangles. Another distinctive characteristic of the organosolv lignins is the partial etherification at benzylic carbon, here specifically observed in the β-O-4′ substructures (Fig. S1–S5†). The degree of benzylic etherification of β-O-4′ units was estimated by HSQC signal integrations at about 63% for the OS reference fractions and 57% OS protected lignin fractions.

For the kraft process, it is well known that aryl ether linkages are targeted to allow extensive depolymerization through cleavage of β-O-4′ bonds, ionization of phenolics and consequently delignification. The cleavage of such lignins yields new phenolic end groups. Furthermore, it was recently established for the same process that aliphatic side chains are degraded to create new reactive sites for direct condensation between aromatic rings in lignin, yielding new types of carbon–carbon linkages.^36,37^ These direct ring to ring condensations cause shifts in the signals of aromatic carbons in the HSQC resulting in heavily overlapped signals in the aromatic region.

Another common structure in native softwood lignin is the β-5′, and its content is higher for the OS protected lignin.

Overall, from the HSQC results, significant structural differences are observed between the three lignin types. The OS protected lignin is distinguished by mainly native bonds with high content of β-O-4′ bonds. The OS reference lignin has low content of native linkages and possesses modified structures resulting from condensation between aliphatic and aromatic carbons. The kraft lignin on the other hand, also has a low content of native linkages but can be distinguished from the OS reference by lower presence of aliphatic side chains in the structure, as well as higher presence of direct carbon–carbon linkages between aromatic rings. The importance of these structural differences in the formation and properties of LNPs will be discussed in another section of this manuscript.

### Distinct differences in content and type of hydroxyl functionalities and the properties of the LNPs prepared from fractionated lignins

**Fig. 3:**
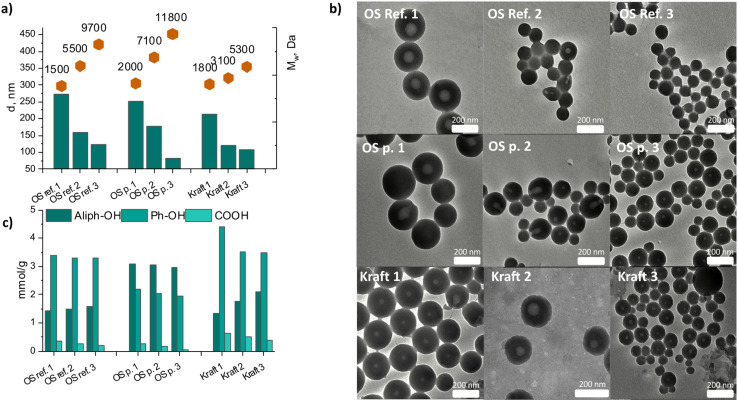
Key trends revealing correlation of LNP inner structure and size to be dependent of *M*_w_ of lignin. Size (diameter) of the LNPs from fractionated lignins *vs*. *M*_w_ of lignin fraction (a), TEM micrographs of the LNPs obtained from fractionated lignins (b) and content of carboxylic groups, aliphatic and phenolic hydroxyls in the lignin fractions (c). Indexes 1, 2, 3 corresponds to soluble, initial and insoluble fractions respectively.

The lignin polymer also contain aliphatic-, carboxylic- and phenolic hydroxyl groups.^32^ These were quantified by ^31^P NMR and are presented in Fig. 3c, Tables S3–S5 and Fig. S32–S37.† The OS protected lignin has a significantly higher content of aliphatic hydroxyls, compared to the OS reference and kraft lignin fractions. This is due to the physical and chemical protection achieved during the extraction of OS protected, which preserve both native inter-unit linkages as and a large part of the aliphatic hydroxyls in lignin. Such protection prevents the production of new phenolic hydroxyls through hydrolysis of β-O-4′ bonds. Hence, the high amount of aliphatic hydroxyls and low amount of phenolic hydroxyls in the OS protected lignin are consistent with the high degree of preservation of the native lignin structure. The lower content of aliphatic OH in OS reference when compared to OS protected, can also be attributed to the higher degree of benzylic etherification in the reference OS. The higher degree of benzylic etherification was verified in the HSQC studies discussed earlier. Contrary to OS protected lignin, the OS reference lignin fractions and the kraft fractions possess lower aliphatic hydroxyl content, higher phenolic hydroxyl content and low content of β-O-4′ bonds.

In general, the size of LNPs was found to decrease with an increase in *M*_w_ of lignin (Fig. 3a). This is consistent with the literature suggesting faster nucleation for higher *M*_w_ lignin which is due to their more hydrophobic character.^40,41^ The role of molecular composition and molecular-weight-dependency in the formation of lignin nanoparticles has been investigated and discussed. Softwood and hardwood kraft lignins were used after stepwise solvent fractionation, aiming to investigate an impact of molecular composition of lignin (syringyl- *vs*. quaiacyl type) and molecular weight on the properties of corresponding LNP's.^35^ In-depth studies on how the macromolecular lignin structure, in terms of inter-unit linkages, affect the properties of LNPs are however still lacking. The differences in the chemical properties of lignin, together with similarities in the molar mass of some of the fractions obtained in this work, offer opportunities for detailed studies on the effects of structure on LNP properties. The yields of the refined fractions are presented in Table S8.†

For similar *M*_w_, *e.g.*, as seen for the ethanol-soluble fractions (Fig. 3a, 1500–2000 Da), the LNPs sizes (Fig. 3a) are in the order OS-Ref-1(272 nm) > OS-protected-1 (252 nm) > kraft-1 (213 nm). This was an interesting observation since no correlation between molecular weight and LNP size is observed, meaning that the observed LNP size differences were probably due to structural differences between the lignin fractions.

According to the TEM analysis (Fig. 3b), all the lignin fractions except OS Ref insoluble gave the LNPs with low-density structures in the middle, which presumably is a hole. The TEM analysis also revealed the presence of Pac-man-like structures in LNPs from initial fractions kraft and OS protected lignin and also in the LNPs from OS protected soluble fraction (Fig. 3b). These structures are possibly due to the LNP formation mechanism, which has been suggested to be micellar^38^ where the elongated lignin micelles,^39^ formed in acetone/water solution, roll up upon acetone evaporation, forming lignin nanostructures. So far there is no explanation why such structures were observed only in certain fractions, but their appearance is probably connected to the molecular lignin structure in solution, *M*_w_ of lignin, lignin concentration, and the number and type of functional units in it.

The LNPs from kraft lignin are significantly smaller than the LNPs from OS lignins. One plausible explanation for this is differences in the hydrophobicity of the lignin molecules which affects the nucleation kinetics when subjected to LNP preparation. The more hydrophobic lignin molecules yield smaller LNPs. Another explanation is the effect of molecular structure on packing density of LNPs. A branched substructure of kraft lignin molecules, due to condensed linkages such as 5–5′ interunit linkage, leads to a more densely packed morphology.^42^ It is therefore plausible that, at similar molecular weight, molecules of kraft lignin in solution have a smaller hydrodynamic radius than those of the two OS lignins, leading to smaller LNPs. A third explanation lies in the higher aromatic density of kraft lignin at the molecular level when contrasted with the other two lignins. This higher density is due to the significant elimination of aliphatic side chains in lignin structure during the kraft pulping process and the formation of new C–C bonds between aromatic rings.^36,43^ The scarcity of hydroxylated side chains in kraft lignins may indeed affect the packing density during LNPs formation.

The ethanol-insoluble fractions gave the lowest LNP size, 122 nm and 107 nm for lignin from OS Ref 3 (insoluble), and the kraft 3 (insoluble) respectively, and 81 nm for OS 3 (insoluble) lignin (Fig. 3a). The observation that OS 3 (insoluble), which had the highest *M*_w_ also forms the smallest particles is consistent with the previously discussed role of *M*_w_ in nucleation kinetics leading to LNP size control. A faster nucleation manifested in higher *M*_w_ lignin yielded smaller LNPs. A discrepancy to this rule is however observed for OS ref insoluble, which had a higher *M*_w_ than its kraft insoluble counterpart but yielded larger LNPs, pointing again toward the impact lignin structure on LNP size.

**Fig. 4:**
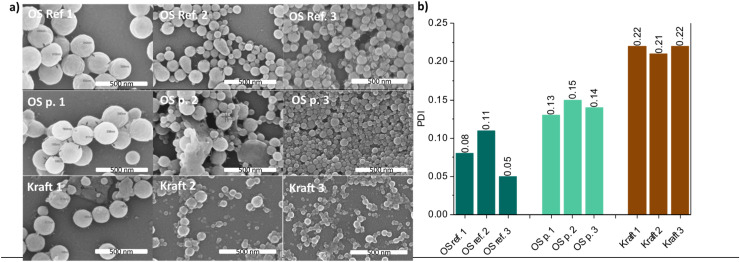
SEM micrographs of the LNPs obtained from fractionated lignins (a) and polydispersity indices (b) for corresponding LNP. Indexes 1, 2, 3 corresponds to soluble, initial and insoluble fractions respectively.

The electron microscopy analysis revealed that all the LNPs are spherical, with some morphological differences depending on the lignin used. All the LNPs from low *M*_w_ fractions possess surface-wrinkled morphology, while the LNPs from EtOH soluble fraction of OS protected lignin contain a hole or a dimple on their surface (Fig. 4a). The LNPs from OS Ref lignin had the lowest polydispersity indices (PDIs), measured by DLS (Fig. 4b). This indirectly suggests high levels of molecular weight homogeneity in this lignin type, since the LNPs size can be related to the *M*_w_ as discussed earlier. The LNPs from kraft lignin possess the highest PDIs values which can be explained by the high level of heterogeneity inherent to kraft lignin. These PDI values were also in agreement with SEM data (Fig. 4a). For instance, the high PDI values of the LNPs from kraft lignin were manifested in the SEM micrographs as a high number of unreacted lignin aggregates. In contrast, the LNPs prepared from OS protected lignin possessed lower PDIs and appeared on the SEM micrographs as uniformly shaped NPs free of aggregates.

Based on our results (Fig. 3) and other literature, it seems evident by now that for a given lignin type (*i.e.*, lignin from a particular source and process), the molecular weight determines the size of LNPs. This is attributed to the nucleation kinetics where higher *M*_w_ lignins nucleate faster and consequently form smaller LNPs. However, when LNPs prepared from lignins from different processes are compared, the LNP size may not directly relate to *M*_w_.

**Fig. 5:**
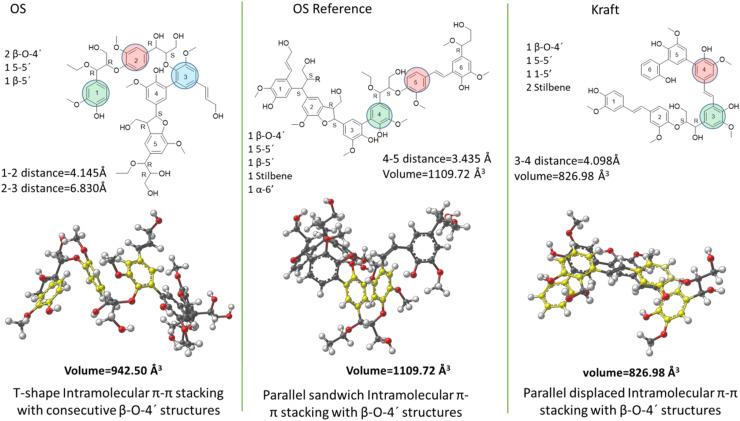
Representative chemical structures and their models for selected lignin fractions revealing their intermolecular self-assembly patterns. Structural motifs of the low-*M*_w_ fractions selected lignins and the inter-unit linkages and their respective lowest energy conformers modelled by DFT in gas phase (hydrogens are omitted for clarity). Units participating in π–π stacking are depicted in yellow.

As earlier stated, we hypothesize that these differences in properties of LNPs are the outcome of the significant variation of chemical structure. The presence of carbohydrates may contribute to some of the LNP properties, however, the carbohydrate contents of the ethanol-soluble fractions were estimated by HSQC and all found to be <0.5%. It is thus assumed this should negligibly affect the LNP properties. Therefore, to study the effect of lignin structure on LNPs, we constructed representative structural motifs for the ethanol soluble fractions of the three lignins using the analytical data obtained through 1D and 2D NMR studies. The ethanol soluble fractions were specifically chosen to study the effect of structure because they had similar molecular weights and therefore effects of molecular weight on the LNP properties are eliminated. The motifs are shown in Fig. 5. The three molecules can be distinguished by certain properties based on their structure. For instance, the OS protected lignin has more flexible (foldable) aryl ether (β-O-4′) linkages than the other two lignins. The kraft lignin on the other hand is very stiff due to higher content of direct coupled aromatic rings in its structure. We reasoned therefore that such differences in molecular properties may lead to conformational differences which may affect LNP properties.

The folding enables intramolecular π–π stacking and has been reported for lignin molecule in D_2_O by Tokunaga *et al*.^44^ In contrast, kraft lignin molecules are stiffer due to carbon–carbon bonds between aromatic rings and are deficient in linkages through flexible aliphatic side chains.

The packing density of lignin molecules during LNPs assembly, as we propose, depends also on the differences in molecular structure and functionality which govern both the core and interphase interaction with water molecules as follows: in all three types of LNPs, the aromatic structures form the core, while the surface is dominated with hydroxyl groups that interact with water molecules. This is consistent with recent ^1^H NMR studies on LNPs.^45^ The simple observation from the ^1^H NMR, showing the dominance of hydrophilic groups on the surface, suggests molecular folding and/or orientation and may be explained by the previously discussed intra- and inter-molecular π–π stacking.

### Density functional theory (DFT) modelling reveals distinct differences in conformational structures

In order to further investigate the role of structure on LNP properties, DFT modelling was applied to the three structural motifs derived from the structural analysis of the ethanol soluble fractions (see Fig. 5 for i.d's) with the aim of revealing structural conformations in selected solvent systems. As discussed in the methods, these results are from a multi-step refinement process starting with a Monte Carlo conformational search, PM6 optimizations of the unique conformations and finally density functional optimizations using the M06-2X method, with the 6-31+G(d,p) basis set and the GD3 empirical dispersion correction. The lowest energy gas phase conformation was subsequently optimized in both water and acetone with the SMD solvation model. In Fig. 5, images from the gas phase modelling are shown for each structural motif.

For all the molecular models, we observe intramolecular π–π stacking with the β-O-4′ inter-unit responsible for the molecular folding. The ethanol soluble OS protected lignin adopts a spiral shape at one end as a result of a T-shape π–π stacking of two neighbouring β-O-4′ rings in the oligomer. The distances between the stacked aromatic rings are 4.9 Å (between rings marked 1 and 2) and 4.7 Å (between rings marked 2 and 3). The other parts of the molecule, due to their stiff character remain relatively linear, but still orient themselves in twisted formations most likely guided by hydrophobic interactions.

The β-O-4′ inter-unit in the ethanol soluble OS reference lignin on the other hand adopts a sandwich type π–π stacking to yield a different conformation. The distance between the stacked rings (rings marked 4 and 5) are 3.4 Å. This distance is typical of sandwich type stacking of benzene dimers, is consistent with distances reported for intra-molecular stacking of aromatic rings in plant cell wall,^46^ and WAX studies related to lignin-based thermosets.^47^

The ethanol soluble kraft lignin molecule forms two sandwich type intramolecular stackings. The distances between the stacked rings are 3.5 Å (rings 1 and 5) and 3.4 Å (rings 2 and 3).

Overall, distinct differences in conformations resulting from structural differences are demonstrated. The volumes of the molecules were measured and found to be in the order OS ref (1109 Å^3^) > OS protected (976 Å^3^) > Kraft (818 Å^3^). The relatively lower volume for kraft lignin molecule is explained by the relative low levels of aliphatic side chains in its structure which allows for denser packing of aromatic rings. This also justifies the observed formation of multiple intramolecular π–π stacking when compared to the other two lignins, where a higher abundancy of aliphatic side chains may set limits for multiple stacking possibilities due to geometrical constrains.

The motifs where then modelled in solvent systems used for the synthesis of LNPs (acetone and water) to investigate if conformational changes occur during LNP assembly. Interestingly, the same molecular conformations were maintained in the modelled solvents, albeit changes in molecular volume (see Fig. S39†). For the OS protected and OS reference samples, a slight shrinkage is observed in water, and a slight expansion in acetone. The kraft lignin remained in principle the same in both solvents. The trend in the volumes observed for the gas phase modelling was also maintained in these solvents, *i.e.*, OS ref > OS protected > kraft. The results suggest that the molecular conformations are maintained during the synthesis of LNPs. Interestingly, the trend in molecular volumes obtained by the DFT studies is consistent with the trend in LNP sizes obtained by experimental SEM studies discussed earlier. This lends credence to the maintenance of molecular conformations in the assembled LNP. The dependence of the size of LNPs on molecular structure of lignin is hereby substantiated. The relatively denser kraft lignin molecular conformation may confer denser packing of the core yielding smaller LNP. This observation is also consistent with the measured surface functionality of LNPs by ^1^H NMR, now discussed.

### ^1^H NMR unfolds the surface structure of LNPs

Solution-state NMR provides information on the superficial structure of NPs, *e.g.*, the overall ligand architecture and its chemical environment^48^ for metal NPs, but applying these methods to polymeric NPs, such as LNPs has not been previously possible. In a recent work by (Pylypchuk *et al.* 2020),^45^ however, a method for successfully applying liquid-state NMR to LNPs to investigate their surface chemistry was developed. The main challenge in applying liquid-state NMR to lignin nanoparticles stems from the fact that signals are so weak that even traces of non-deuterated solvent will make the peaks from LNPs unreadable. This problem was solved by using a water suppression program combining Presaturation with Excitation sculpting using dual Watergate blocks, which was able to remove the solvent peak completely without distorting the spectra, thus allowing the LNP peaks to be measured.^45^ This method already gave insights into surface chemical interactions of LNPs with cancer cells^49^ and also was applied to the LNPs in the present work. For simplification, we focused only on LNPs from ethanol soluble (low *M*_w_) fractions.

The NMR studies of the LNPs were performed in D_2_O to preserve the original structure of the LNPs in water. To compare the signal of original lignin in solution with the spectra of their LNPs suspension, ^1^H NMR of the lignin fractions dissolved in DMSO-d_6_ was performed as a reference. The ^1^H NMR spectra of the unfractionated lignins are included in Fig. S11–S19.† The full-range integrated ^1^H spectra of LNPs are presented in Fig. S20–S28.†

**Fig. 6:**
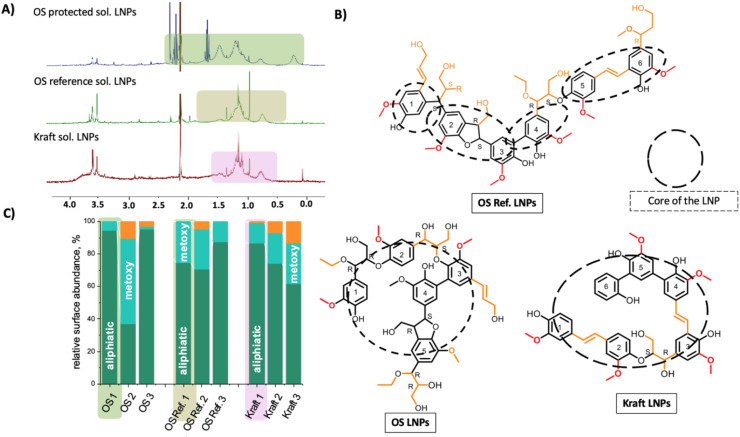
Liquid-state ^1^H NMR confirms the superficial layer structure of LNPs in aqueous solutions based on the predicted lignin macromolecule model. ^1^H NMR spectra for the LNPs prepared from soluble lignin fractions (a), proposed structures on the surface of LNP from soluble fractions (b), distribution of main units over the surface of LNPs as estimated from ^1^H NMR (c). Indexes 1,2,3 corresponds to soluble, initial and insoluble fractions respectively.

In the NMR spectra of LNPs from soluble fractions presented in Fig. 6a, the aromatic protons show a weak signal, while the signals from aliphatic protons (5 to 0 ppm) were more pronounced, suggesting the presence of aliphatic lignin groups, located above the surface of the LNPs and interacting with the solvent. For all the LNPs from all lignin fractions, the signal at 3.3–3.8 ppm can be attributed to the methoxy groups in the G-units of lignin, which are “sticking out” of the surface of LNPs. The low-intensity singlets at about 2.72 ppm can be attributed to β-protons in β–β structures. In softwood LNPs, the methoxy group signals in G-units may overlap those of β-protons in phenylcoumaran structures and/or γ-protons in γ-hydroxylated β-O-4′ substructures in the 3.4–3.8 ppm region. The strong signals observed at 1.8–0.04 ppm can be attributed to the non-oxygenated aliphatics. The non-oxygenated aliphatics are typically not common in lignin structure and likely originate from methyl protons in ethanol residues. The complete removal of ethanol from lignin fractions during recovery practically impossible, detected by ^31^P NMR as narrow signal appearing between 146–147 ppm in the aliphatic region (Fig. S36 and S37†). The partial ethoxylation at benzylic carbon, observed in the HSQC studies could in part contribute to the H NMR signals between 1.8–0.04 ppm.

In a simplification, we distinguish three main chemical groups of the protons in lignin NMR spectra, namely aromatic protons (7.5–6.5 ppm), protons associated with methoxy groups (3.8–3.3 ppm) and non-oxygenated aliphatic units (1.8–0.04 ppm). Comparing the ratio between those units, as shown in Fig. 6c, one can come up with very interesting conclusions. For the LNP samples (Fig. 6c), the aromatic protons are almost invisible (except for some fractions, as shown in Fig. S20, 22–23, S27 and S28†), along with decreased methoxy group signals intensities, when compared to molecular lignin. Furthermore, the content of the non-oxygenated aliphatic units in LNPs significantly increased. Thus, we have depicted the most abundant chemical groups as shown for the soluble fractions in Fig. 6c. This allowed reconstructing of the chemical structure of LNPs from soluble fractions, as it appears from the liquid state ^1^H NMR and 2D NMR of lignin fractions in a molecular state. The proposed surface structure is presented in Fig. 6b. Interestingly, the NMR deciphered surface structure of LNP is consistent with the surface structure of the individual molecules studied by DFT modelling.

There is some correlation between the content of the functional units in the initial lignin and the composition of the LNPs surface. For instance, OS protected lignin possesses the highest non-oxygenated aliphatic content, and this is reflected in the NMR spectra of corresponding LNPs, where the signals at 1.8–0.04 ppm were the strongest, Fig. S29 and Table S7.† Both OS lignins do have some incorporation of ethanol through covalent interaction at the benzylic carbon of lignin. The aliphatic hydroxyl contents in the OS lignins are also higher. These hydroxyls are located on flexible aliphatic chains. Phenolic hydroxyls are the dominant functionality feature in the kraft lignin fractions. It is therefore reasonable for the kraft lignin that methoxy groups from G-units which are by design in close proximity to phenolic hydroxyls dominate on the surface of these LNPs, while the signal of aliphatic is depleted (Fig. 6b). In fact, as earlier discussed, the kraft lignin fraction is scarce in aliphatic side chains and has a high aromatic density at the molecular scale due to the abundance of direct ring-to-ring coupling. The high content of non-oxygenated aliphatics on the surface of ethanol soluble kraft LNPs is likely due to residual ethanol that interact with the lignin through physical forces.

Another noticeable feature, observed only for LNPs from this lignin fraction, is a relatively strong doublet of doublets at 7.66–7.23 ppm, which can be attributed to the protons of coumaryl aldehyde and/or to C_β_ protons of stilbene β-5′structures. Their appearance in the spectra indicates that these aromatic units are present on the surface of LNPs. The signal of aromatic protons of different intensities was observed also for the LNPs from OS Ref at 8.39–8.31 ppm and signals at 7.09–6.18 ppm were observed for LNPs obtained from the soluble and insoluble fractions of kraft lignin.

The general conclusion of the ^1^H NMR study is that the surface of the LNPs from OS protected lignin is more aliphatic, compared to LNPs from OS Ref and kraft lignin. The high content of aliphatic groups on the surface of LNPs from OS protected lignin agrees with aliphatic content in initial lignin and most probably dominated by aliphatics linked in β-O-4′ sub-structures. Also, it is possible to assume that signals from C_α_ and C_β_ protons of β-O-4′ linkages were suppressed together with water and are not seen in the spectra.

**Fig. 7:**
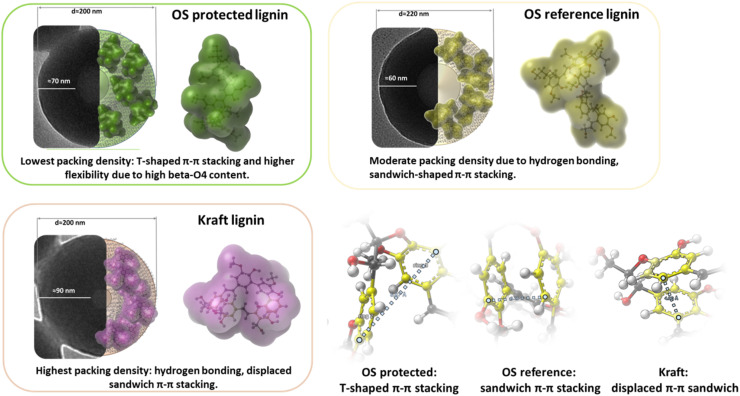
The shape and size of LNPs are dependent on the intermolecular self-assembly of the lignin macromolecule, which is a key driver in the LNP formation. In the figure: three TEM images with inserted molecular spheres. Besides the images are enlargements of the spheres the conformed molecules. To the bottom right are representative intramolecular π–π stacking observed for the respective lignins.

The visual perception of all the three molecular models already shows tendencies of an inner core of aromatic rings and surfaces containing methoxyl groups, aliphatic- and aromatic-hydroxyls, and reflected in Fig. 7.

## Conclusions

The impact of lignin structure and the content of functional groups on the size, morphology and surface properties of corresponding LNPs was investigated and validated using DFT modelling calculations. The variations in the chemical structure of investigated lignin samples was a result of the extraction procedures, where kraft process and two ethanol-based organosolv processes were used. State-of-the art NMR was performed to establish distinct structural differences between the three lignins, as required for the investigation.

The polydispersity indices are shown to be smaller for LNPs from organosolv lignins, while kraft lignin, yielded more polydisperse LNPs.

To address the role of molecular structure without interference from molecular weight effects, on LNPs properties, we identified three lignin fractions of comparable molecular weight. These were then used to synthesize LNPs and distinct properties were obtained which could be related to molecular structure.

Structural motifs for the 3 refined lignins having similar molecular weight were created from the analytical data and used in DFT modelling calculations to further investigate the role of lignin structure in the formation of LNPs. It was demonstrated that flexible inter-unit linkages in lignin, specifically the β-O-4′ substructures, yield molecular folding resulting to intra-molecular π–π stacking. This demonstrated the role of molecular structure of lignin in conformational differences of the lignins. The type of π–π stacking was either sandwich or T-shaped type, depending on the exact molecular structure. DFT modelling further showed these molecular conformations do not change in the solvent systems used for LNP synthesis, meaning that the shapes are maintained during the molecular assembling to form LNPs.

^1^H NMR analysis of LNPs revealed distinct differences in the surface chemistry of LNPs that were consistent with observations from DFT modelling. The surface of LNPs prepared from OS protected lignin consists mainly of aliphatic residues, with a very small presence of methoxy groups from G-units. The surface of LNPs from kraft lignin alongside methoxy groups from G-units retain a noticeable amount of the aromatic protons, which are weakly interacting with the solvent. The conformational differences established by DFT were also in agreement with LNP morphological studies by TEM, and showed good correlation with LNP size studies by SEM.

Overall, the present work unravels the relationship between molecular structure of lignin and LNP properties, offering a means to molecularly tailor LNPs properties for specified applications.

## Author contributions

Conceptualisation by O.S. and Ma.L. Original draft writing: I.P. with M.K. and Ma.L. Editing manuscript: all authors. Experimental work and data analysis: I.P, M.K., Ma.L., P.L, Theoretical Modelling and related analysis T.E., Ma.L. Funding aquisition: Ma.L., O.S., Mi.L., T.E. All authors have read and agreed to the published version of the manuscript.

## Data availability

All data generated or analysed during this study are included in this published article and its ESI files.† Additional information is available from the corresponding author on reasonable request.

## Conflicts of interest

Maria Karlsson and Martin Lawoko are co-founders of Proligreen AB, a company that produces biorefinery lignin.

## Supplementary Material

GC-025-D3GC00703K-s001
