# Supplementary material for: Molecular understanding of the morphology and properties of lignin nanoparticles: unravelling the potential for tailored applications
Source: Green Chem. 2023 May 12;25(11):4415–28. doi: 10.1039/d3gc00703k (PMC10243429; doi:10.1039/d3gc00703k)
Supplement: GC-025-D3GC00703K-s001 [file GC-025-D3GC00703K-s001.pdf]

Electronic Supplementary Material (ESI) for Green Chemistry.
This journal is © The Royal Society of Chemistry 2023

Supporting information

Molecular understanding of the morphology and properties of lignin nanoparticles: Unravelling the potential for tailored applications

Ievgen V. Pylypchuk[1§], Maria Karlsson[2§], Pär A. Lindén[2], Mikael Lindström[1,2], Olena Sevastyanova[1,2] and Martin Lawoko[1,2*]*

Contents

1. HSQC 2
1.1. OS protected lignin 2
1.2. OS reference lignin 4
1.3. Kraft lignin 6
1.4. Diagnostic shifts for HSQC assignment 8
1.5. Semi quantification of interunit linkages for the different type of lignins 8
2. ^{31}P NMR 9
2.1. Hydroxyl-functionalities for the different lignin 9
3. Size Exclusion Chromatography (SEC) 10
3.1. Molecular weight and dispersity index of lignin fractions 10
4. ^{1}H NMR 10
5. **NMR of LNPs** 16
6. Scheme of biorefinery processes 22
6.1. OS protected lignin 22
6.2. OS reference lignin 22
7. 31P NMR spectra of ethanol fractions 23
8. Extraction and ethanol fractionation yield 28
9. Lignin structures 28
10. DFT: Molecular volume 29

1. HSQC

1.1. OS protected lignin

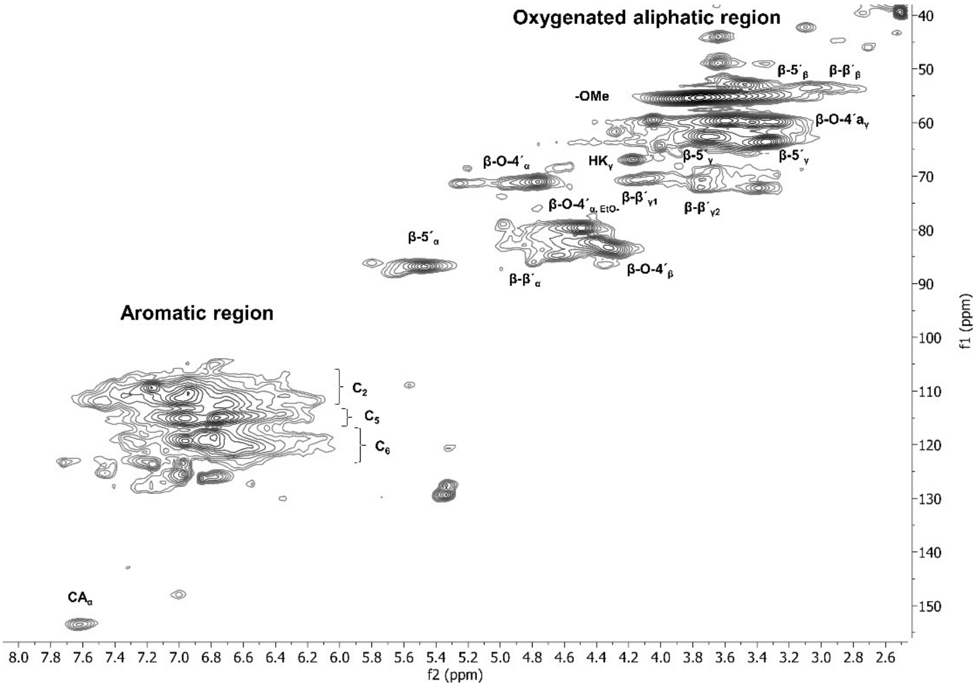

Figure S1. The OS protected lignin. f2 correspond to the ^{1}H and f1 to the ^{13}C dimension, respectively.

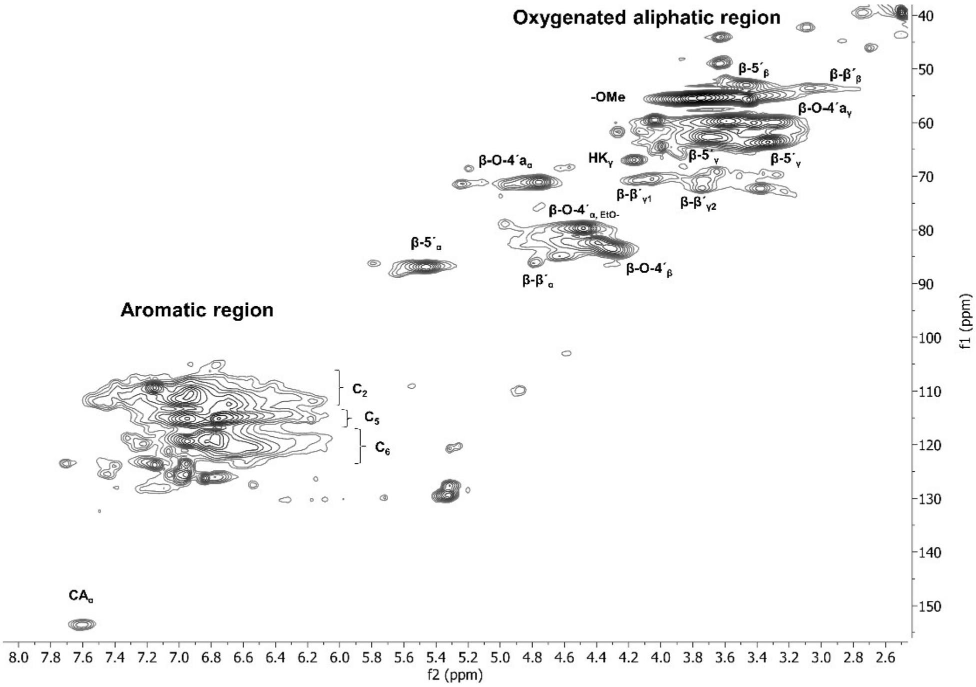

Figure S2. The ethanol soluble part of the fractionated OS protected lignin. f2 correspond to the ^{1}H and f1 to the ^{13}C dimension, respectively.

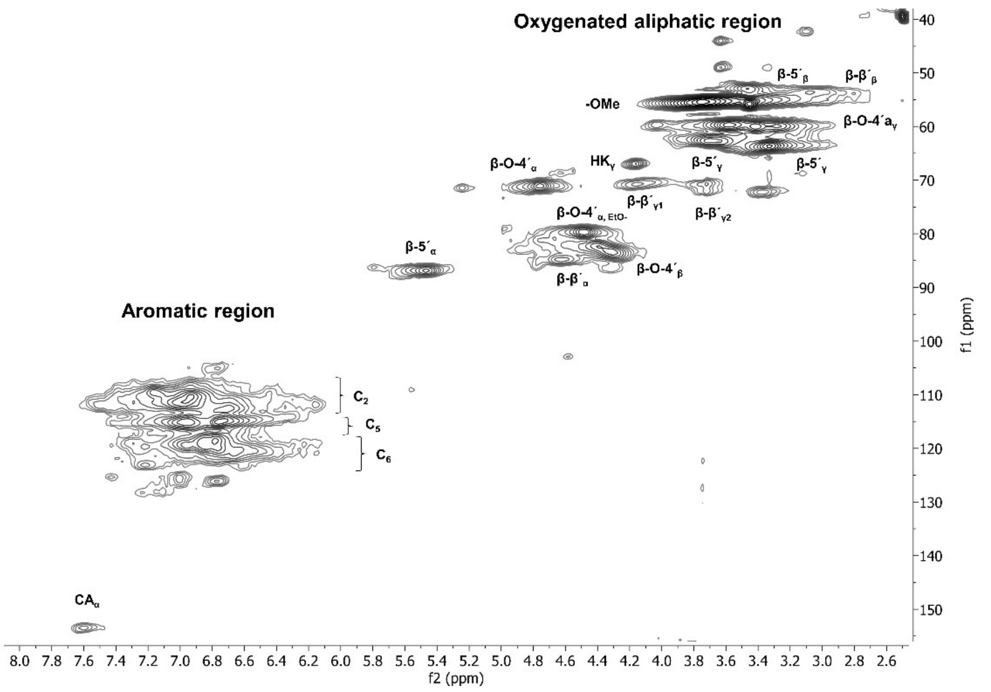

Figure S3. The ethanol insoluble part of the fractionated OS protected lignin. f2 correspond to the ^{1}H and f1 to the ^{13}C dimension, respectively.

1.2. OS reference lignin

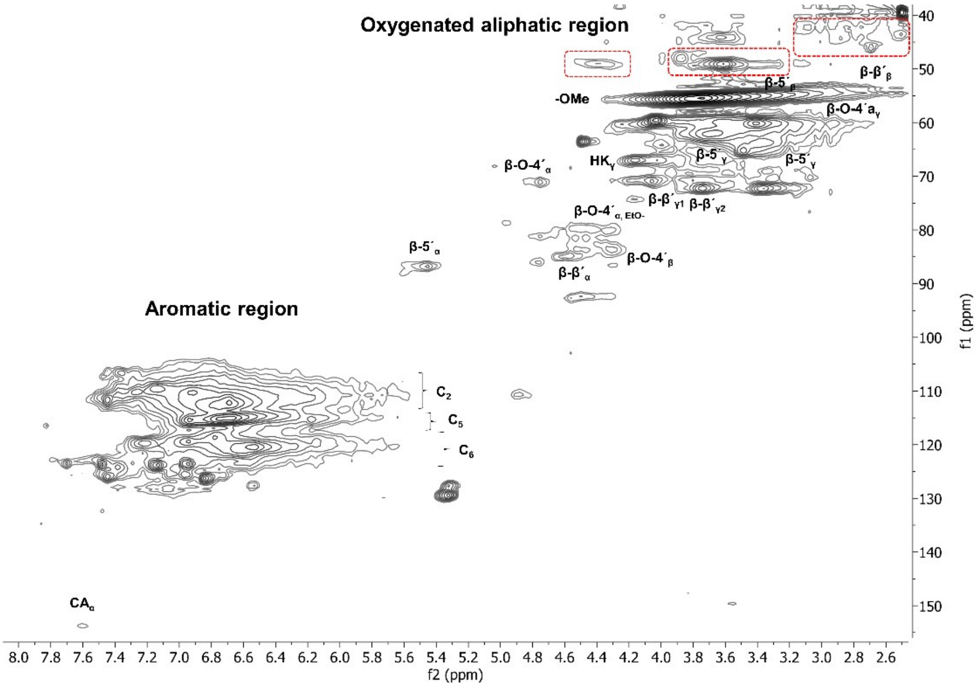

Figure S4. OS reference lignin. f2 correspond to the ^{1}H and f1 to the ^{13}C dimension, respectively. The signals from condensations between the aliphatic sidechain and aromatic ring is marked with red dotted rectangles.

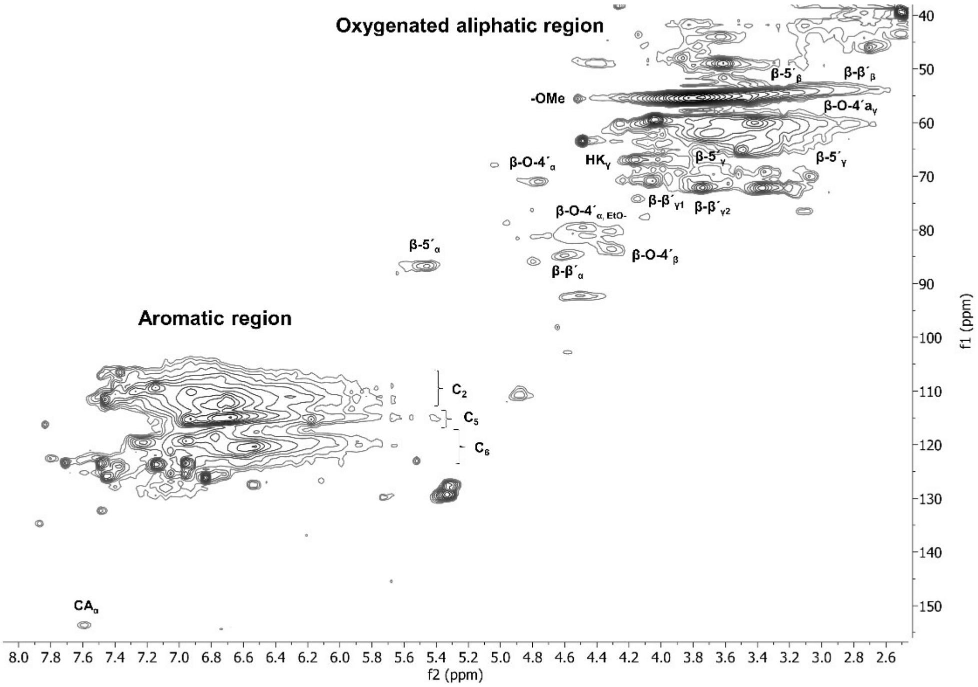

Figure S5. The ethanol soluble part of the fractionated OS reference lignin. f2 correspond to the ^{1}H and f1 to the ^{13}C dimension, respectively.

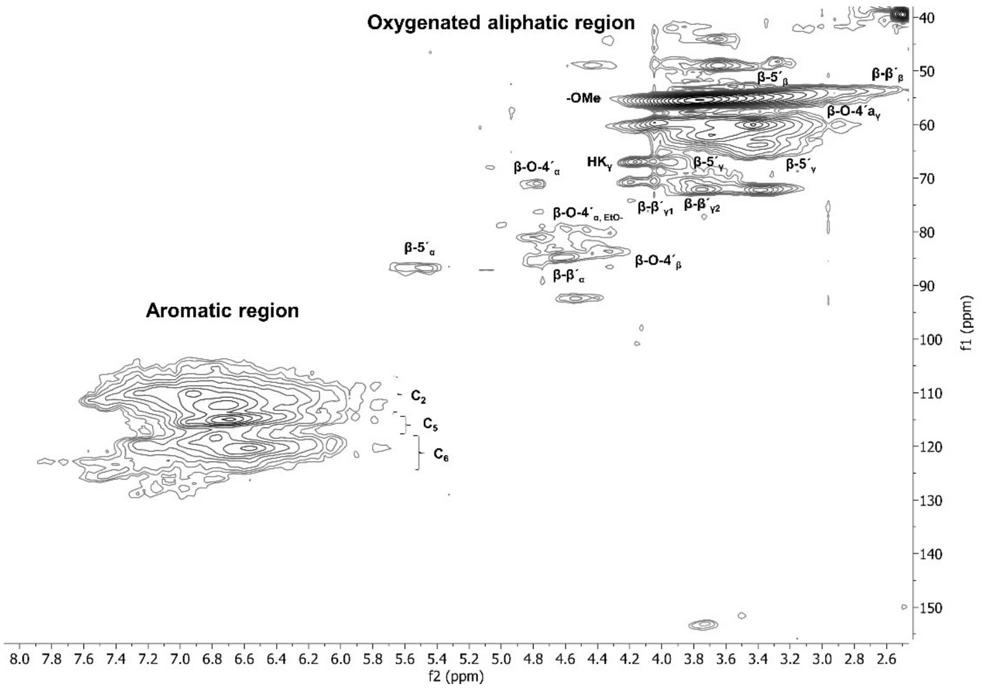

Figure S6. The ethanol insoluble part of the fractionated OS reference lignin. f2 correspond to the ^{1}H and f1 to the ^{13}C dimension, respectively.

1.3. Kraft lignin

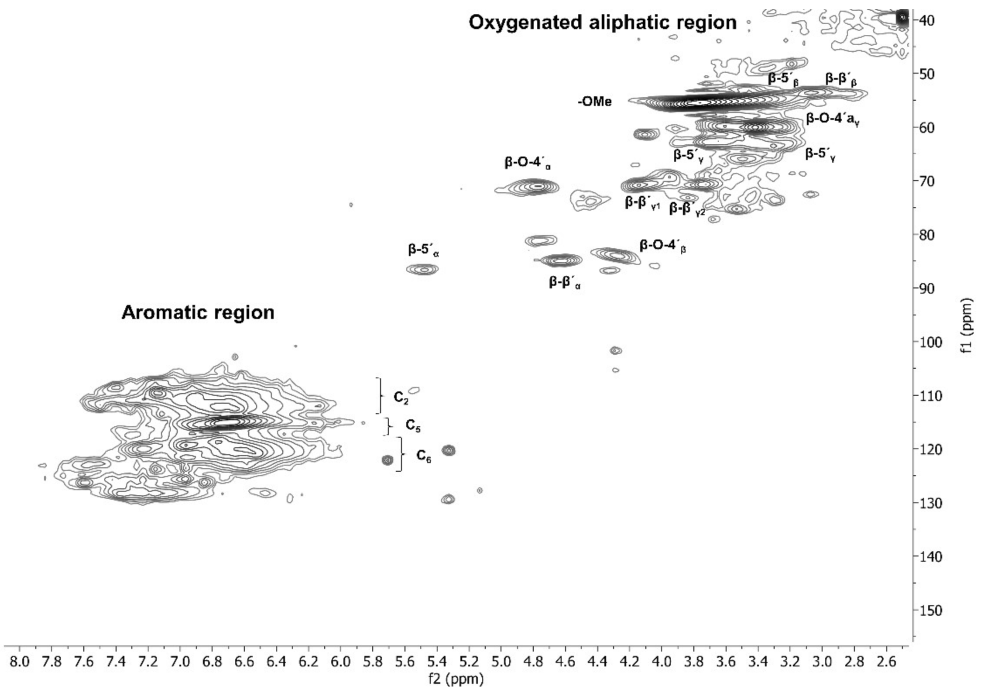

Figure S7. The kraft lignin. f2 correspond to the ^{1}H and f1 to the ^{13}C dimension, respectively.

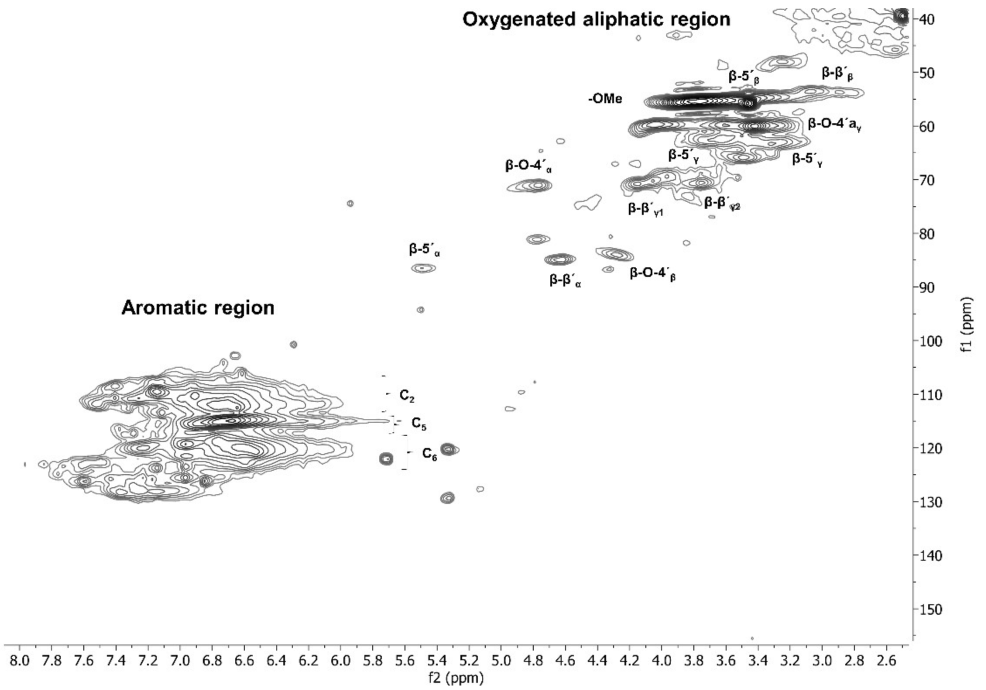

Figure S8. The ethanol soluble part of the fractionated kraft lignin. f2 correspond to the ^{1}H and f1 to the ^{13}C dimension, respectively.

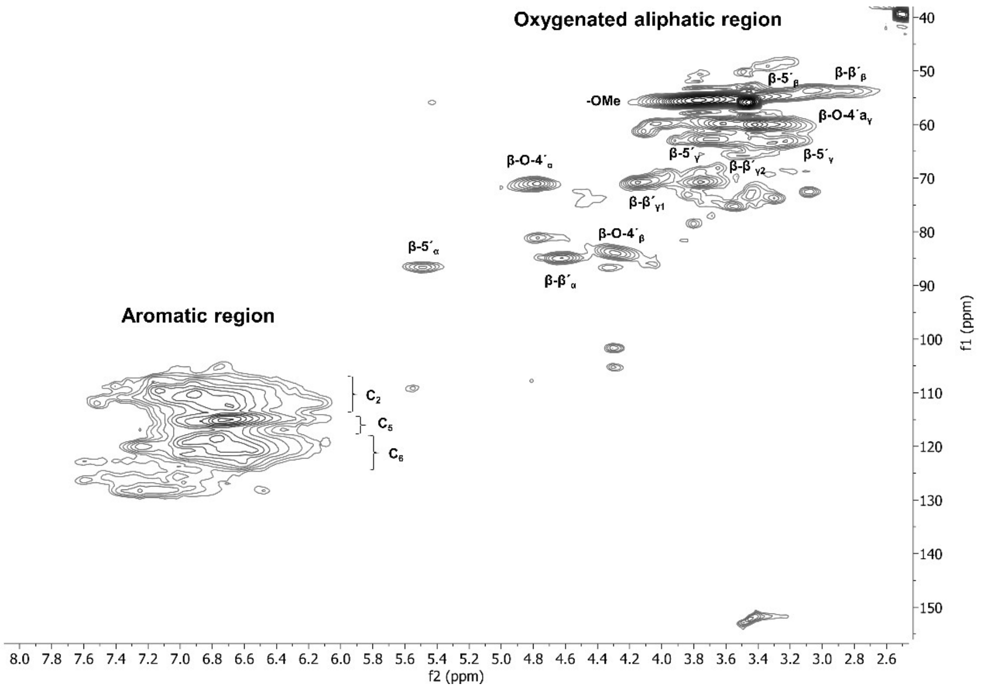

Figure S9. The ethanol insoluble part of the fractionated kraft lignin. f2 correspond to the ^{1}H and f1 to the ^{13}C dimension, respectively.

1.4. Diagnostic shifts for HSQC assignment

Table S1. Chemical shifts for integration of HSQC NMR spectra for inter-unit linkages.

Chemical shifts	C2	β-O-4′, C_α	β-O-4′, Et-C_α	β-O-4′, C_β	β-5′, C_α	β- β ′, C_α
**^{1}H**	7.8-6.1 (7.8-5.6)[1]	5.4-4.4	4.7-4.0	4.6-3.9	5.8-5.0	4.8-4.5
**^{13}C**	113.2-106.1 (113.2-101.9)[1]	74.0-69.0	81.4-76.9	86.6-80.5	91.0-83.2	86.4-83.1

[1]Larger integration of the C2 area for the 2h reference lignin and LignoBoost.

Chemical shifts	Stilbene, β-1′, C_α	Stilbene, β-5′, C_β	Coumarylaldehyde, C_α	HK, C_γ
**^{1}H**	127.8-124.4	121.8-117.8	155.5-151.7	68.5-65.3
**^{13}C**	7.1-6.9	7.5-7.1	7.7-7.4	4.3-4.0

1.5. Semi quantification of interunit linkages for the different type of lignins

Table S2. Semi quantification of interunit linkages.

Lignin	β-O-4′	β-5′	β-β′	Stilbene β-1′, C_α	Stilbene β-5′, C_β	Coumarylaldehyde, C_α	HK, C_γ
**OS protected initial**	32	12	2.0	3.8	3.9	2.0	1.5
**OS protected EtOH soluble**	33	12	1.7	5.6	3.4	2.1	1.4
**OS protected EtOH insoluble**	33	12	2.9	2.3	4.9	2.1	1.7
**OS reference initial**	5.5	2.4	0.63	2.0	4.0	0.29	1.3
**OS reference EtOH soluble**	5.1	2.4	0.48	2.2	4.0	0.32	1.3
**OS reference EtOH insoluble**	3.9	3.3	0.87	1.0	3.9	0.25	1.2
**Kraft initial**	6.8	1.7	1.4	2.9	6.2	n.d.	n.d.
**Kraft EtOH soluble**	3.6	1.0	0.86	3.2	6.0	n.d.	n.d.
**Kraft EtOH insoluble**	9.0	2.9	2.2	2.4	4.5	n.d.	n.d.

2. ^{31}P NMR

2.1. Hydroxyl-functionalities for the different lignin

Table S3. Hydroxyl-functionality of the OS protected lignin.

	**Aliphatic OH**	**C_5–substituted OH**	**Guaiacyl OH**	***p*-hydroxy phenyl OH**	**Carboxylic acid OH**
**Chemical shift**	149.1-145.1	144.7-141.1	140.6-138.8	138.2-137.3	136.6-133.6
**OS protected lignin**	mmol/g	mmol/g	mmol/g	mmol/g	mmol/g
Initial	3.04	0.663	1.31	0.0572	0.166
EtOH soluble	3.09	0.578	1.56	0.0629	0.257
EtOH insoluble	2.95	0.738	1.14	0.0625	0.0625

Table S4. Hydroxyl-functionality of the OS reference lignin.

	**Aliphatic OH**	**C_5–substituted OH**	**Guaiacyl OH**	***p*-hydroxy phenyl OH**	**Carboxylic acid OH**
**Chemical shift**	149.1-145.1	144.7-141.1	140.6-138.8	138.2-137.3	136.6-133.6
**OS reference**	mmol/g	mmol/g	mmol/g	mmol/g	mmol/g
Initial	1.50	1.28	1.88	0.149	0.252
EtOH soluble	1.42	1.11	2.15	0.121	0.350
EtOH insoluble	1.57	1.45	1.68	0.159	0.193

Table S5. Hydroxyl-functionality of the kraft lignin.

	**Aliphatic OH**	**C_5–substituted OH**	**Guaiacyl OH**	***p*-hydroxy phenyl OH**	**Carboxylic acid OH**
**Chemical shift**	149.1-145.1	144.7-141.1	140.6-138.8	138.2-137.3	136.6-133.6
**Kraft**	mmol/g	mmol/g	mmol/g	mmol/g	mmol/g
Initial	1.76	1.28	2.03	0.209	0.508
EtOH soluble	1.34	1.50	2.71	0.200	0.616
EtOH insoluble	2.11	1.54	1.77	0.165	0.376

3. Size Exclusion Chromatography (SEC)

3.1. Molecular weight and dispersity index of lignin fractions

Table S6. Molecular weight and dispersity of the lignin fractions.

Lignin	Mn [g/mol]	Mw [g/mol]	Mz [g/mol]	Đ
OS protected, initial	1800	7100	32000	3.88
OS protected, EtOH soluble	1100	2000	3200	1.83
OS protected, EtOH insoluble	3900	11800	42800	3.05
OS reference, initial	1500	5500	19000	3.68
OS reference, EtOH soluble	800	1500	2400	1.78
OS reference, EtOH insoluble	3200	9700	27600	3.06
Kraft, initial	1270	3100	7200	2.46
Kraft, EtOH soluble	970	1800	3200	1.90
Kraft, EtOH insoluble	2300	5300	9900	2.30

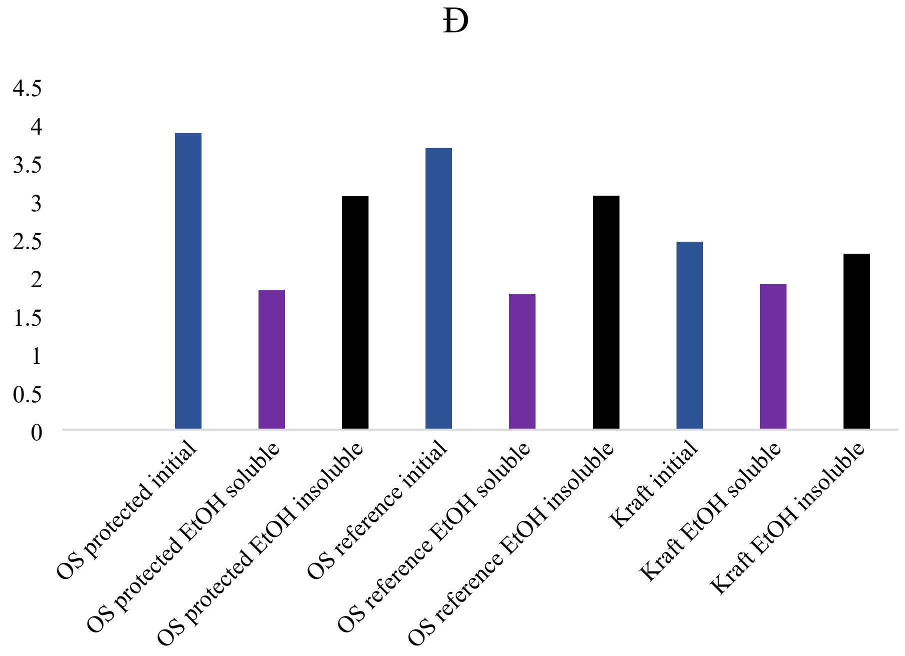

Figure S10. Dispersity index trends for the lignin fractions.

4. ^{1}H NMR

^{1}H NMR of the unfractionated lignins, analysed in DMSO-d6.

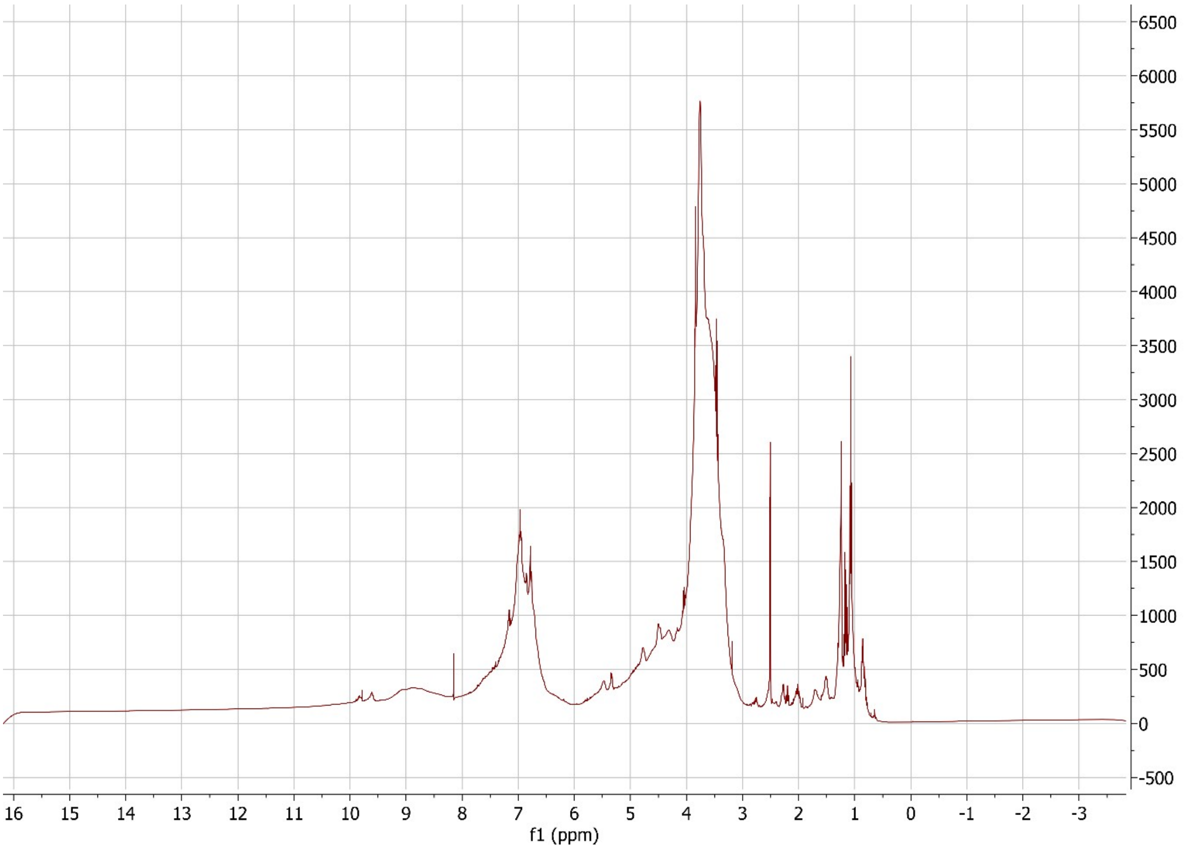

Figure S11. OS protected lignin, initial fraction

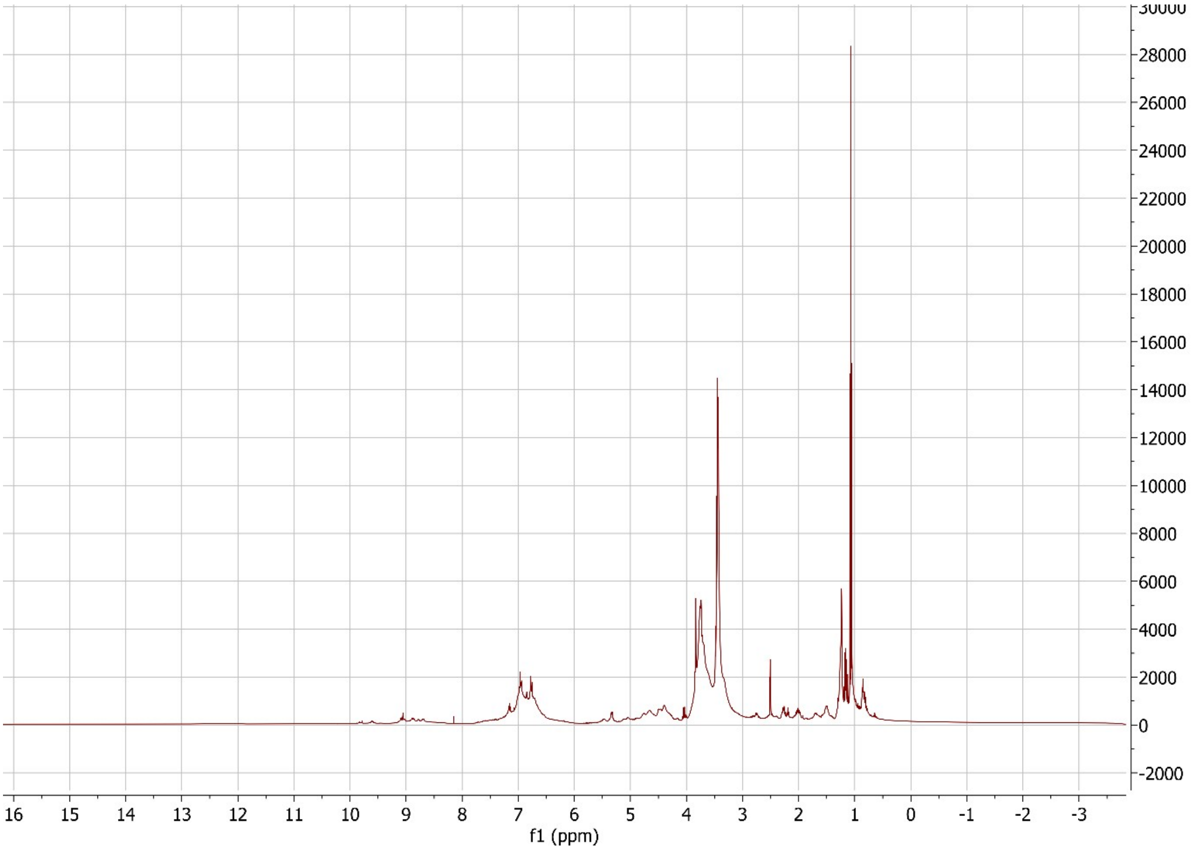

Figure S12. OS protected lignin, ethanol soluble fraction

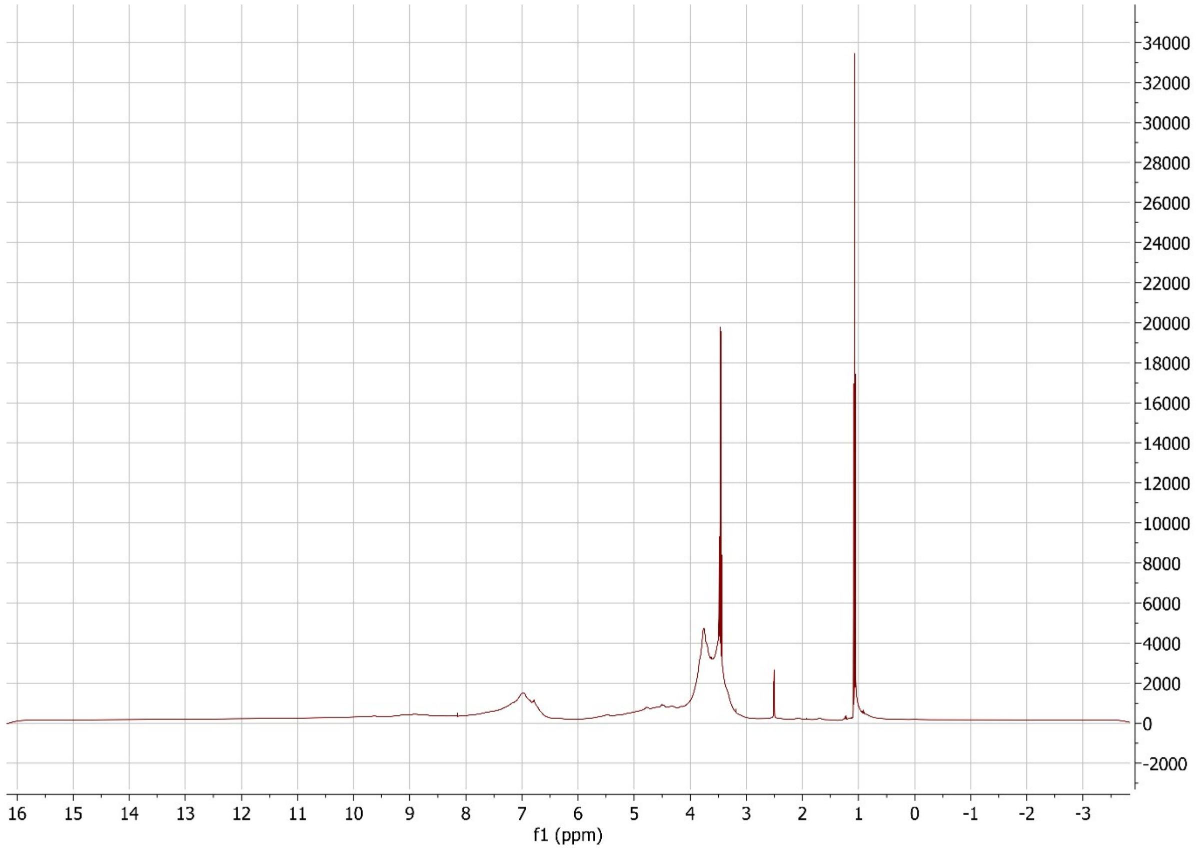

Figure S13. OS protected lignin, ethanol insoluble fraction

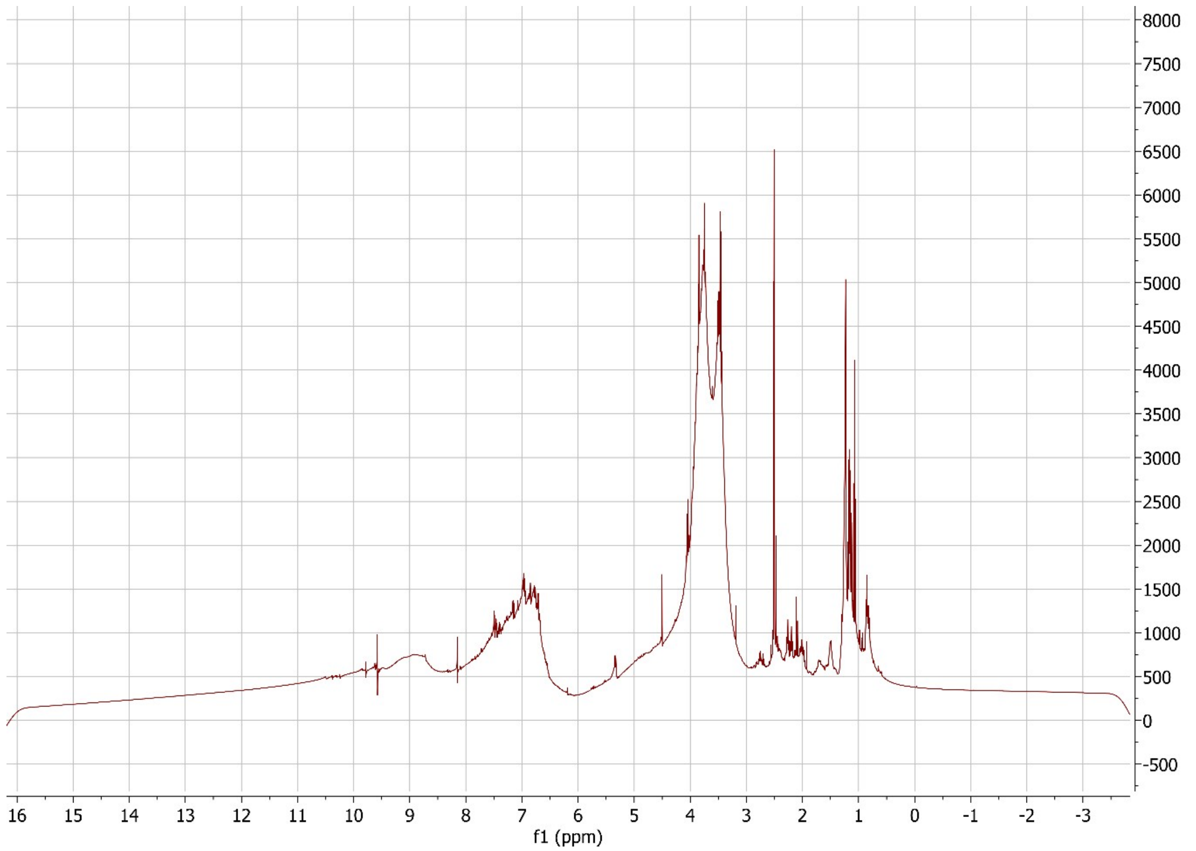

Figure S14. OS reference lignin, initial fraction

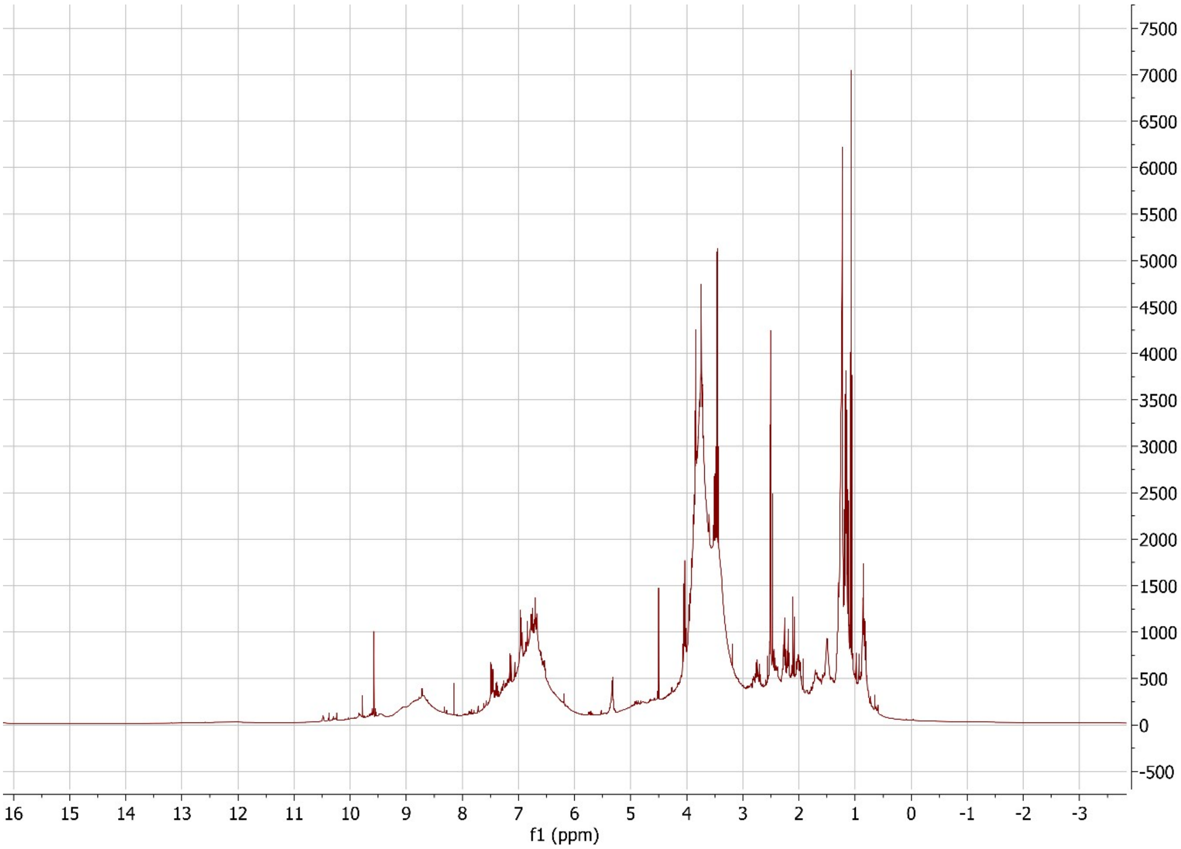

Figure S15. OS reference lignin, ethanol soluble fraction

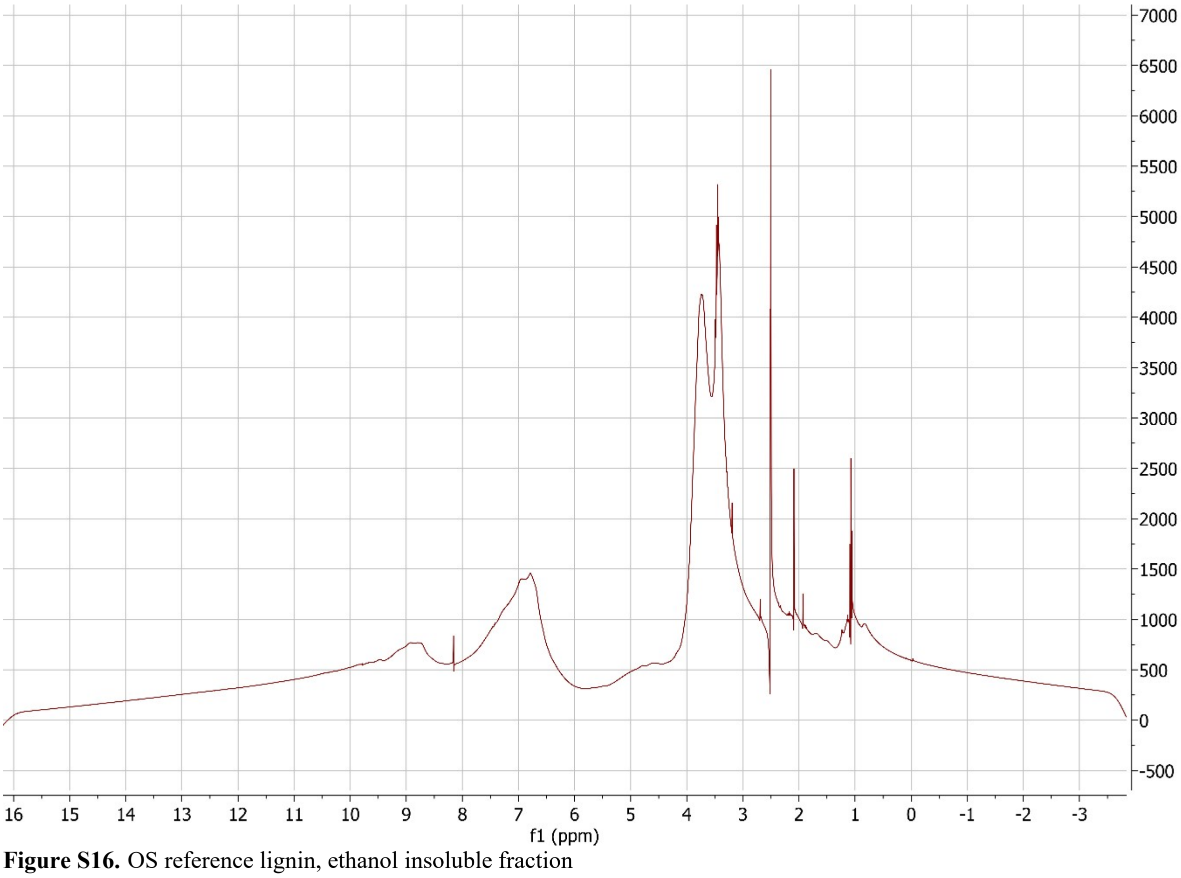

Figure S16. OS reference lignin, ethanol insoluble fraction

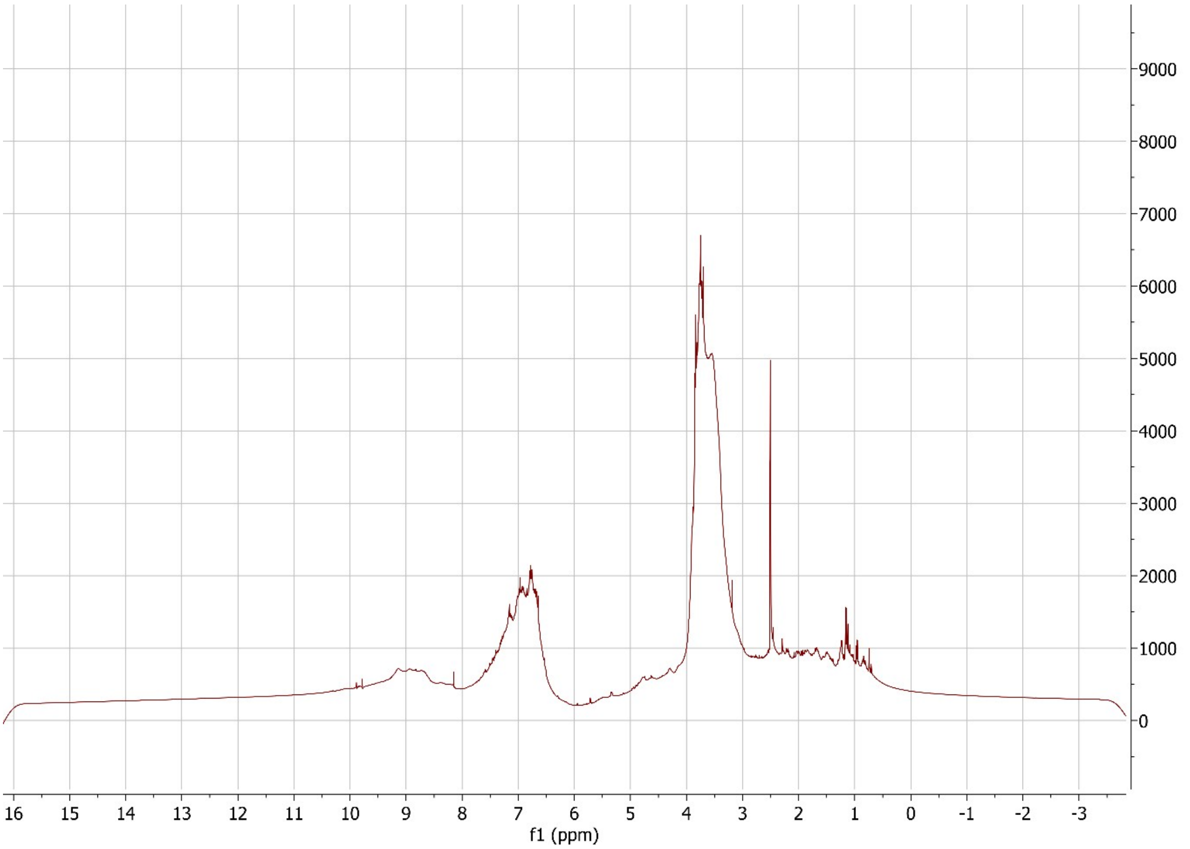

Figure S17. Kraft lignin, initial fraction

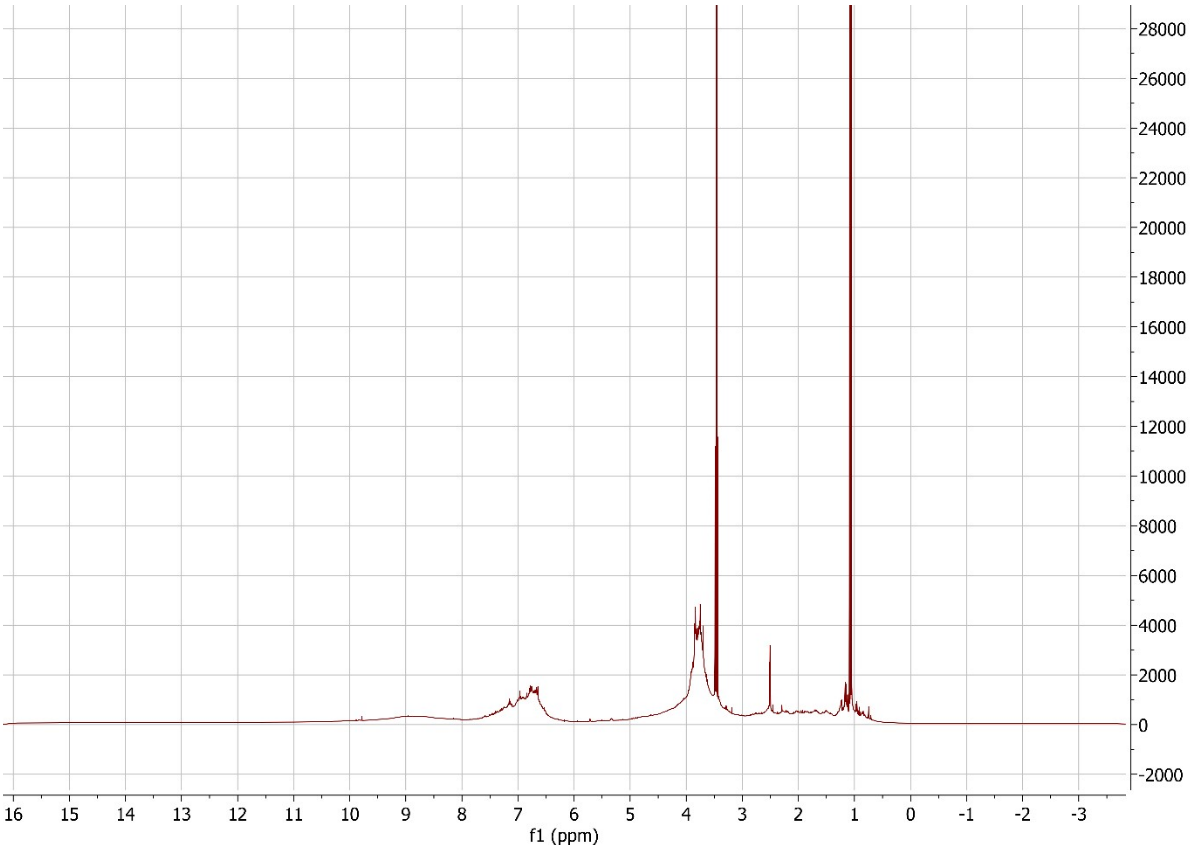

Figure S18. Kraft lignin, ethanol soluble fraction

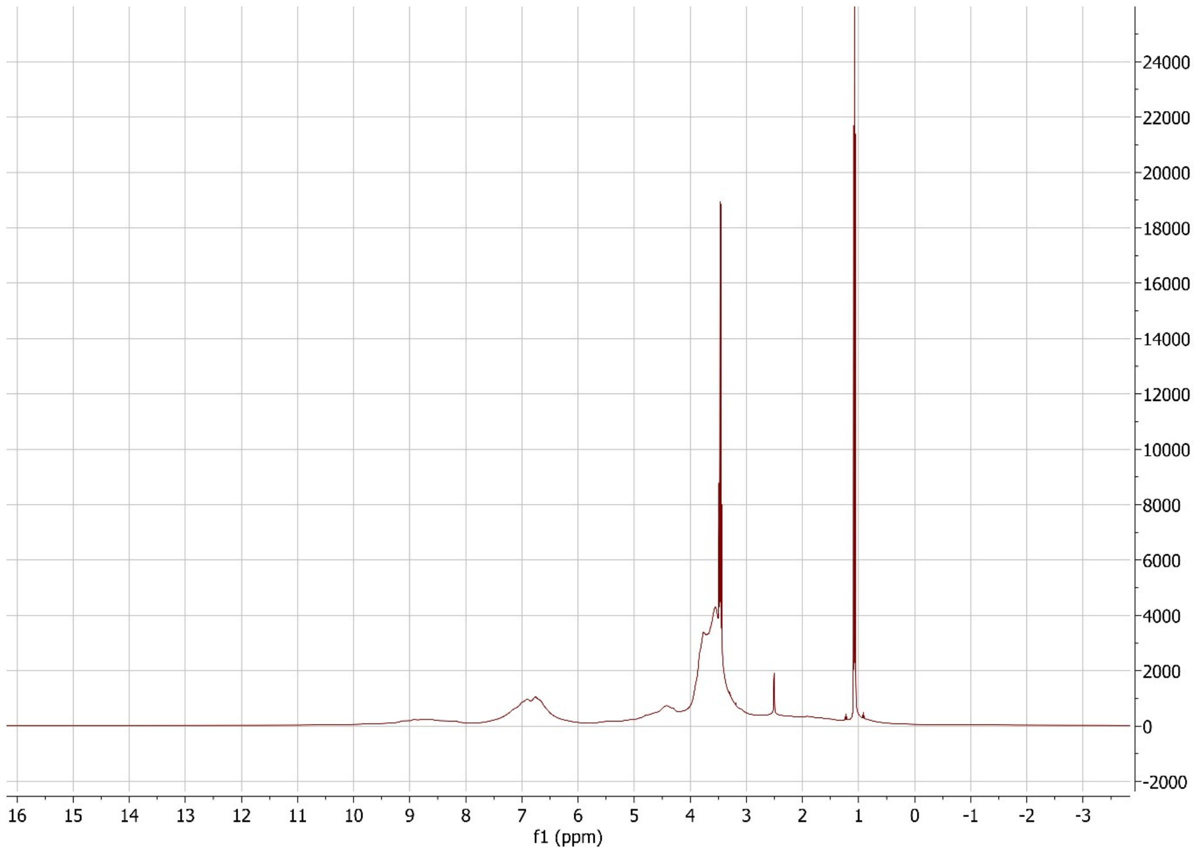

Figure S19. Kraft lignin, ethanol insoluble fraction

5. NMR of LNPs

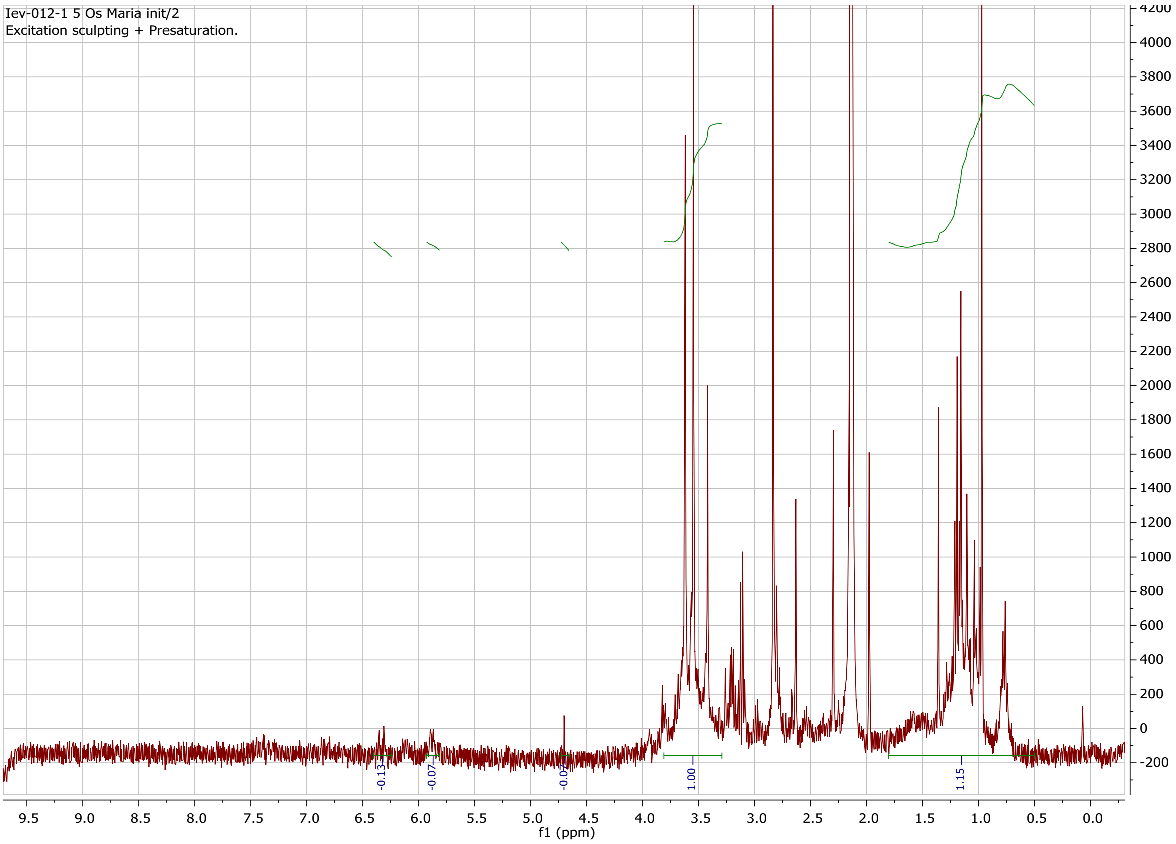

Figure S20. LNPs from OS protected lignin, initial fraction.

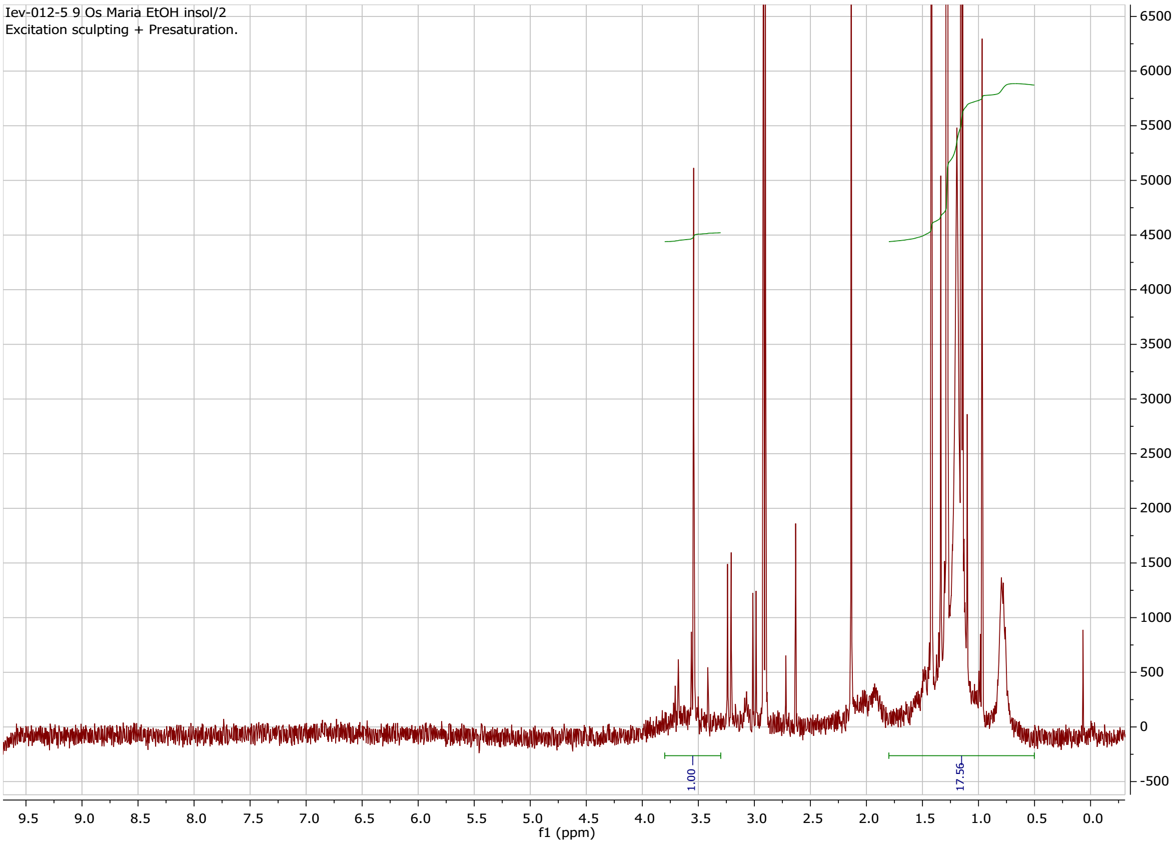

Figure S21. LNPs from OS protected lignin, ethanol insoluble fraction.

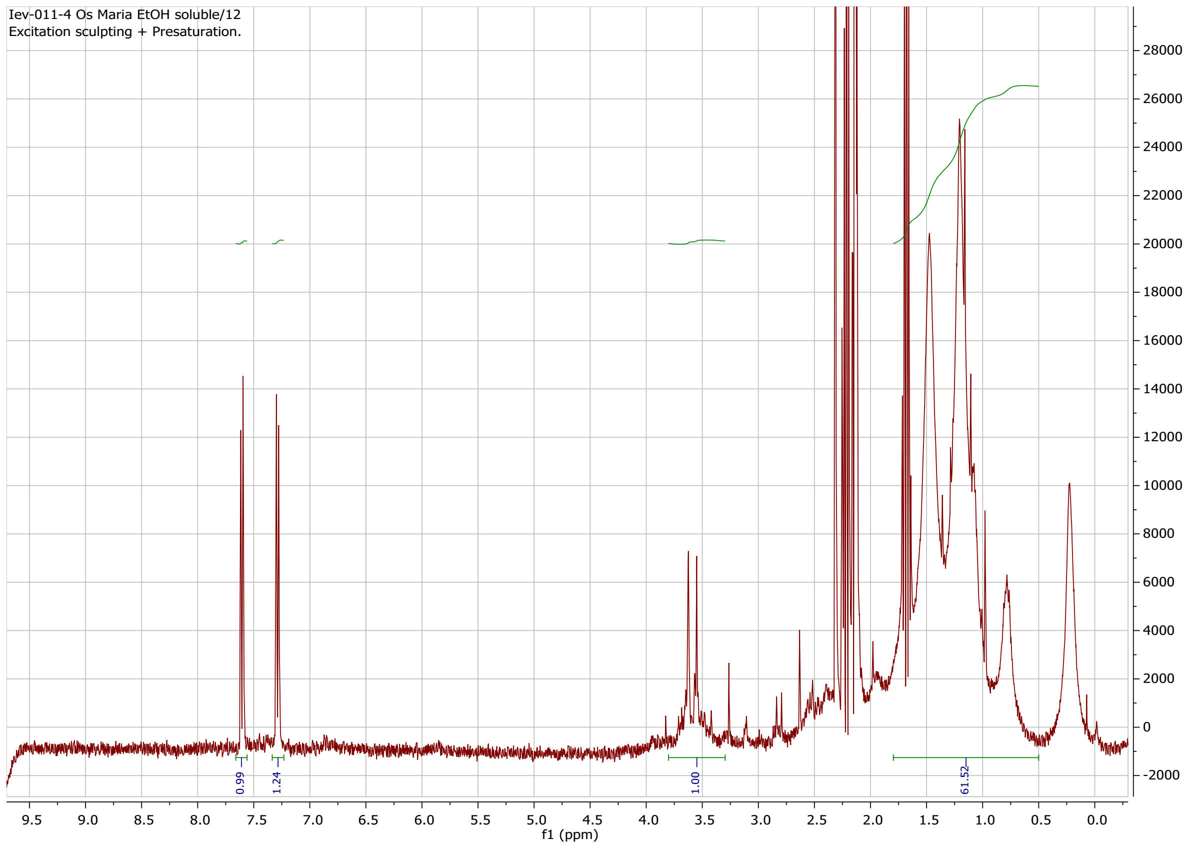

Figure S22. LNPs from OS protected lignin, ethanol soluble fraction.

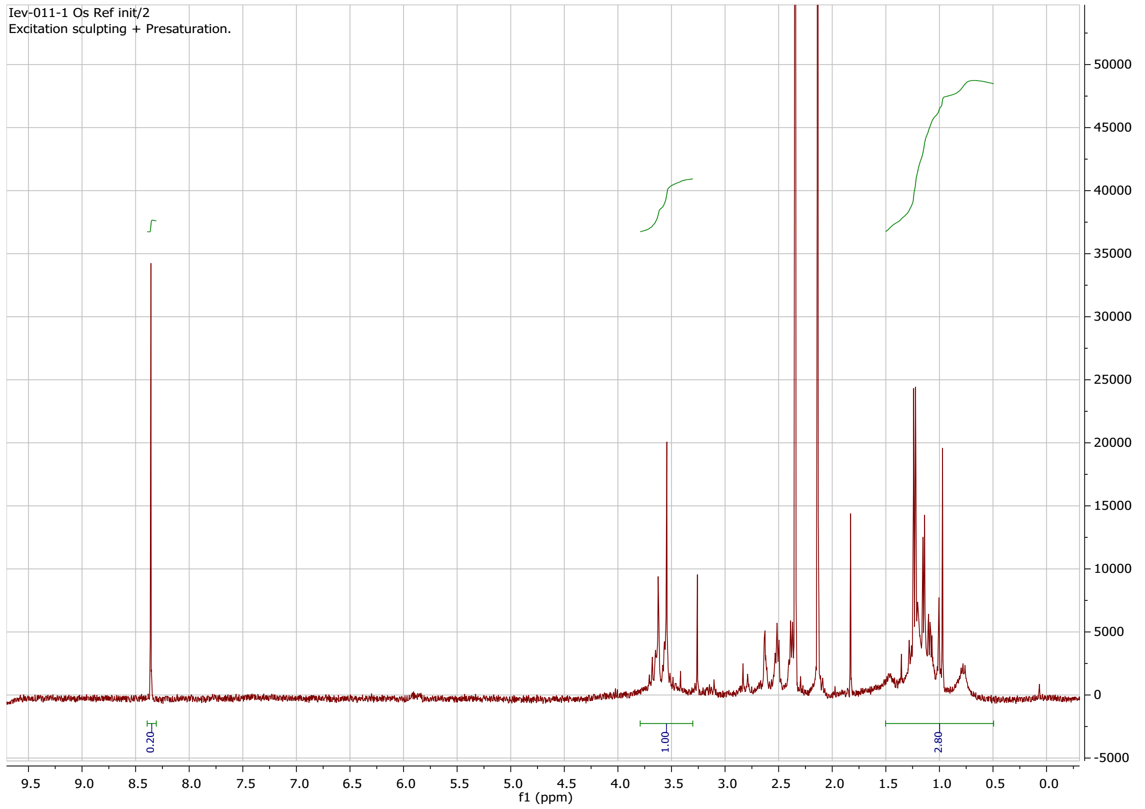

Figure S23. LNPs from OS reference lignin, initial fraction

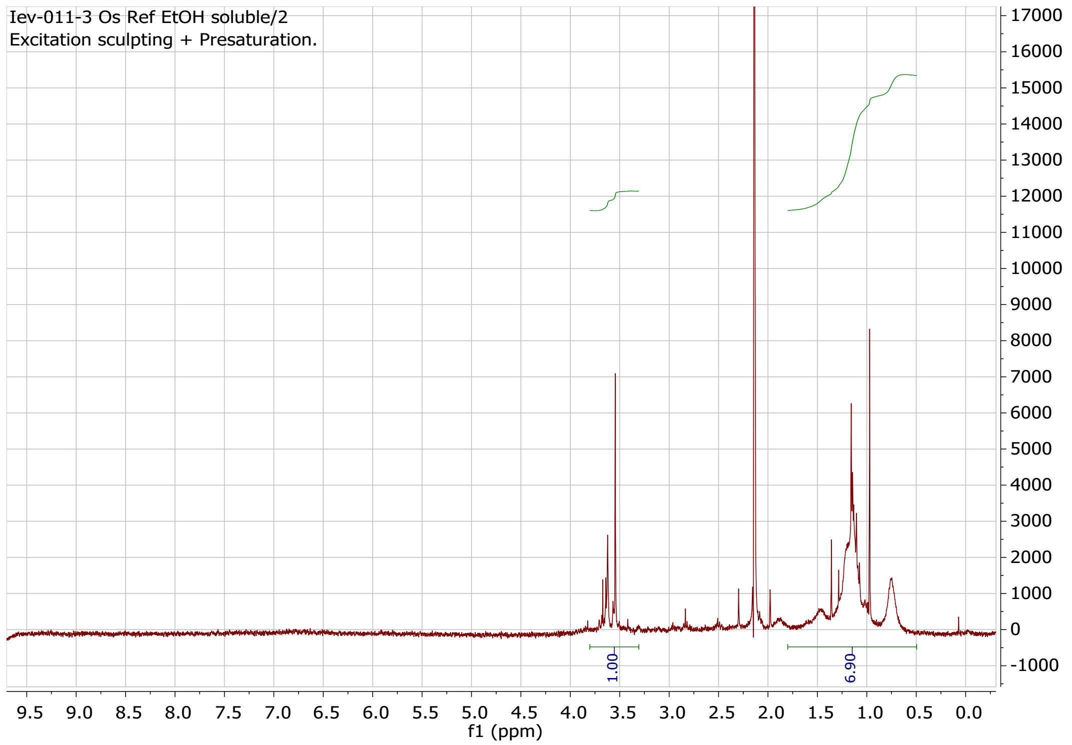

Figure S24. LNPs from OS reference lignin, ethanol soluble fraction.

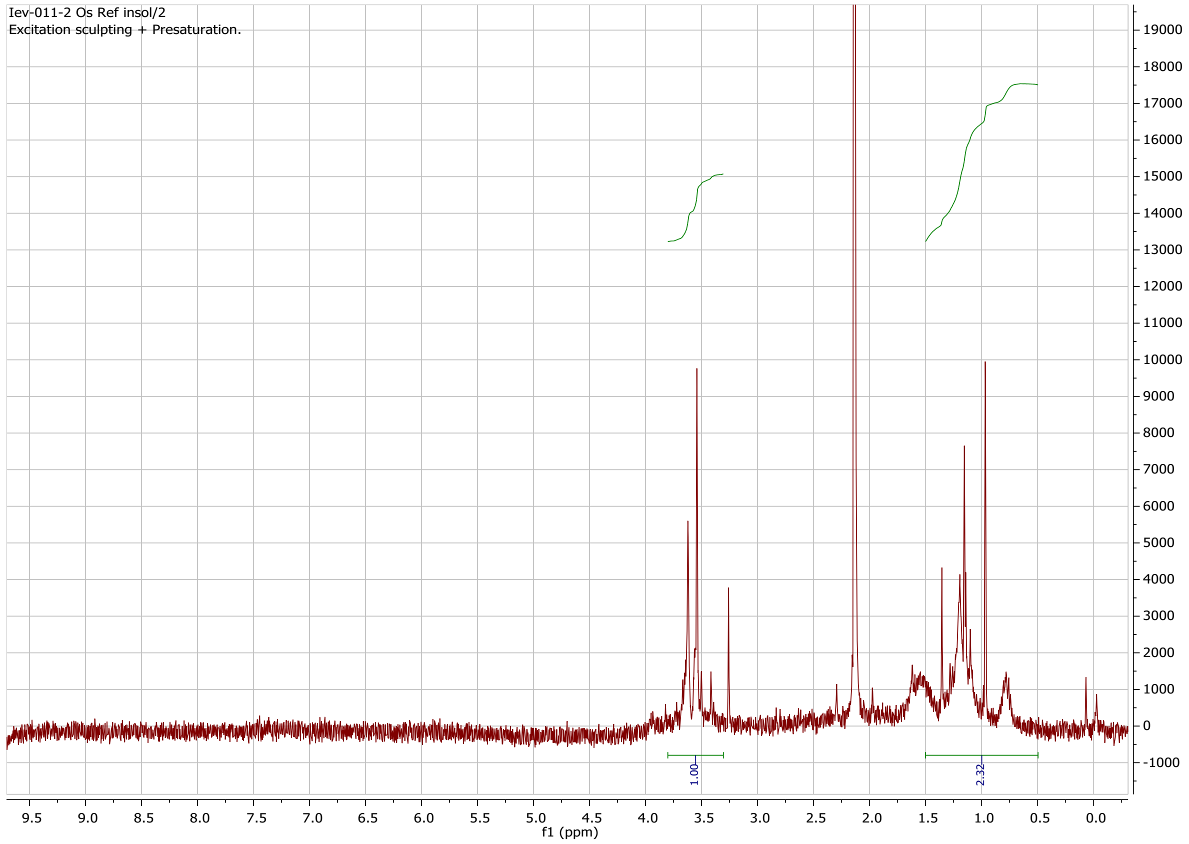

Figure S25. LNPs from OS reference lignin, ethanol insoluble fraction.

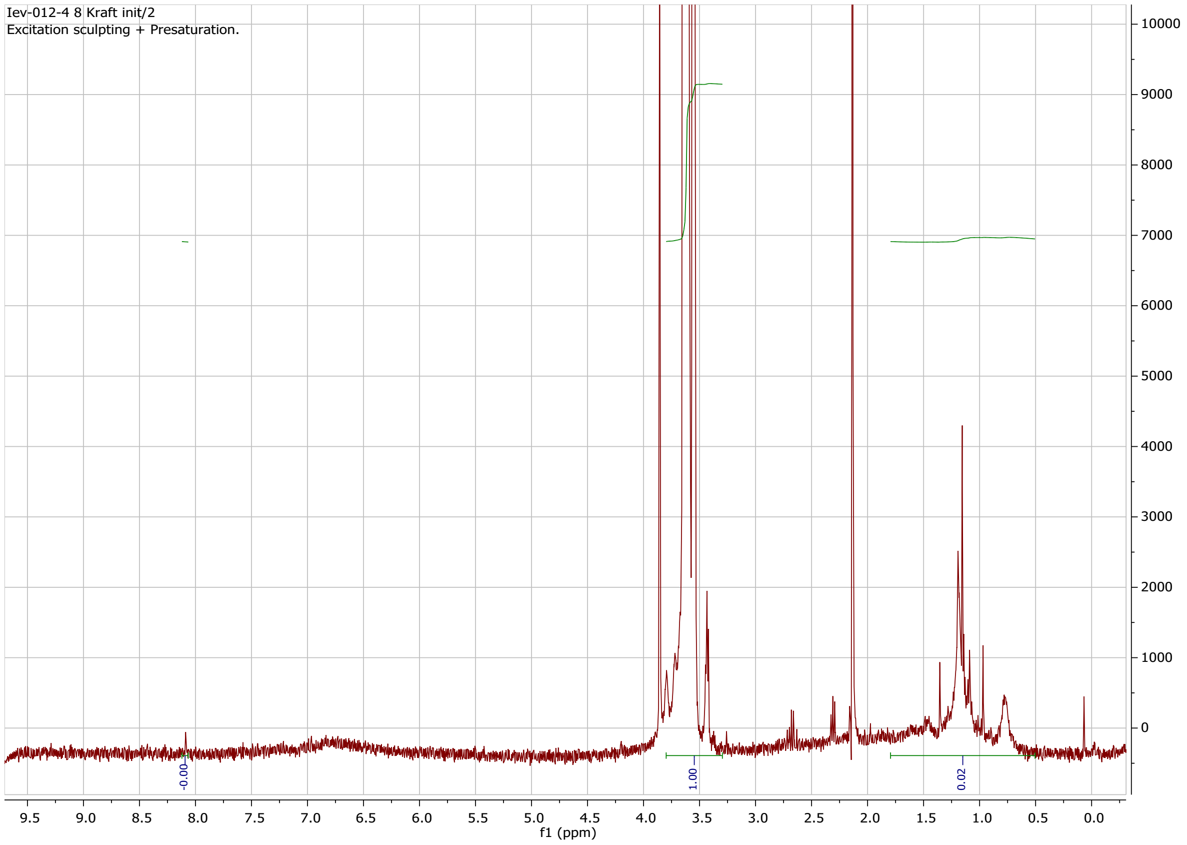

Figure S26. LNPs from kraft lignin, initial fraction.

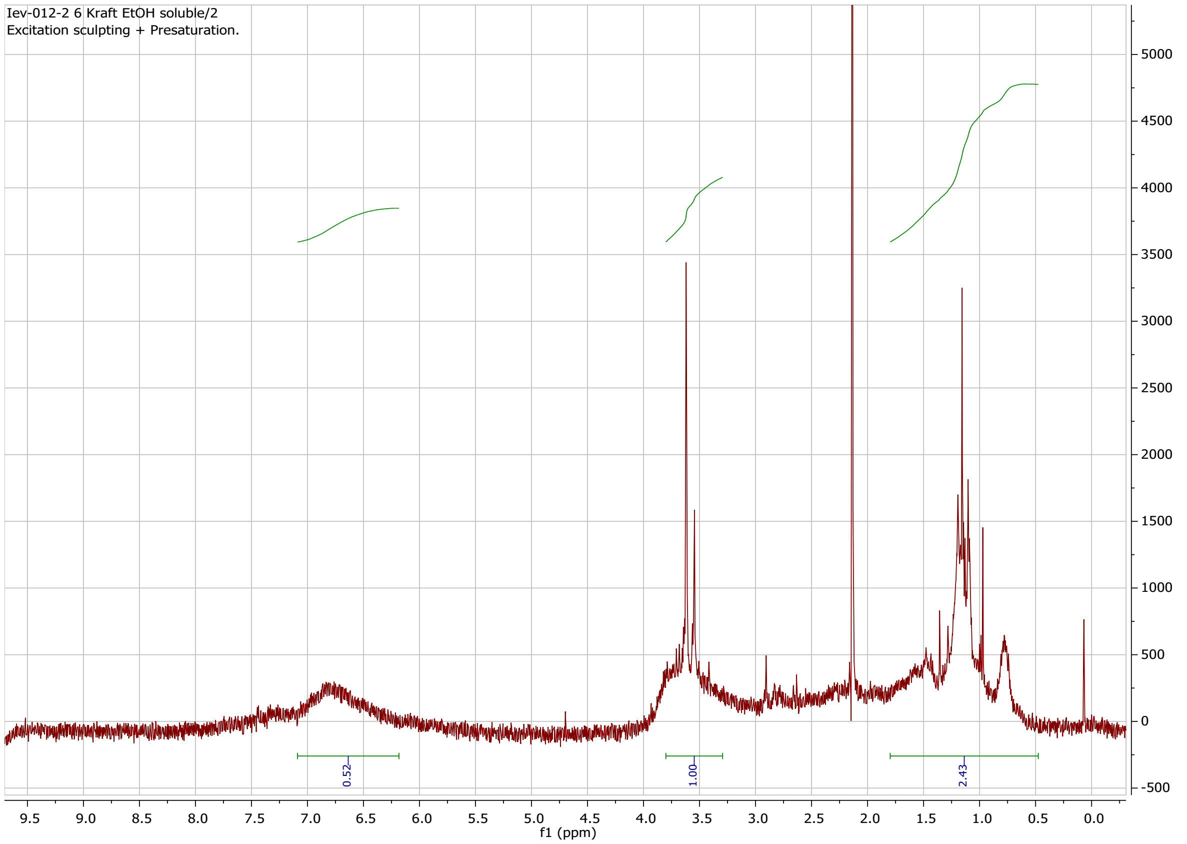

Figure S27. LNPs from kraft lignin, ethanol soluble fraction.

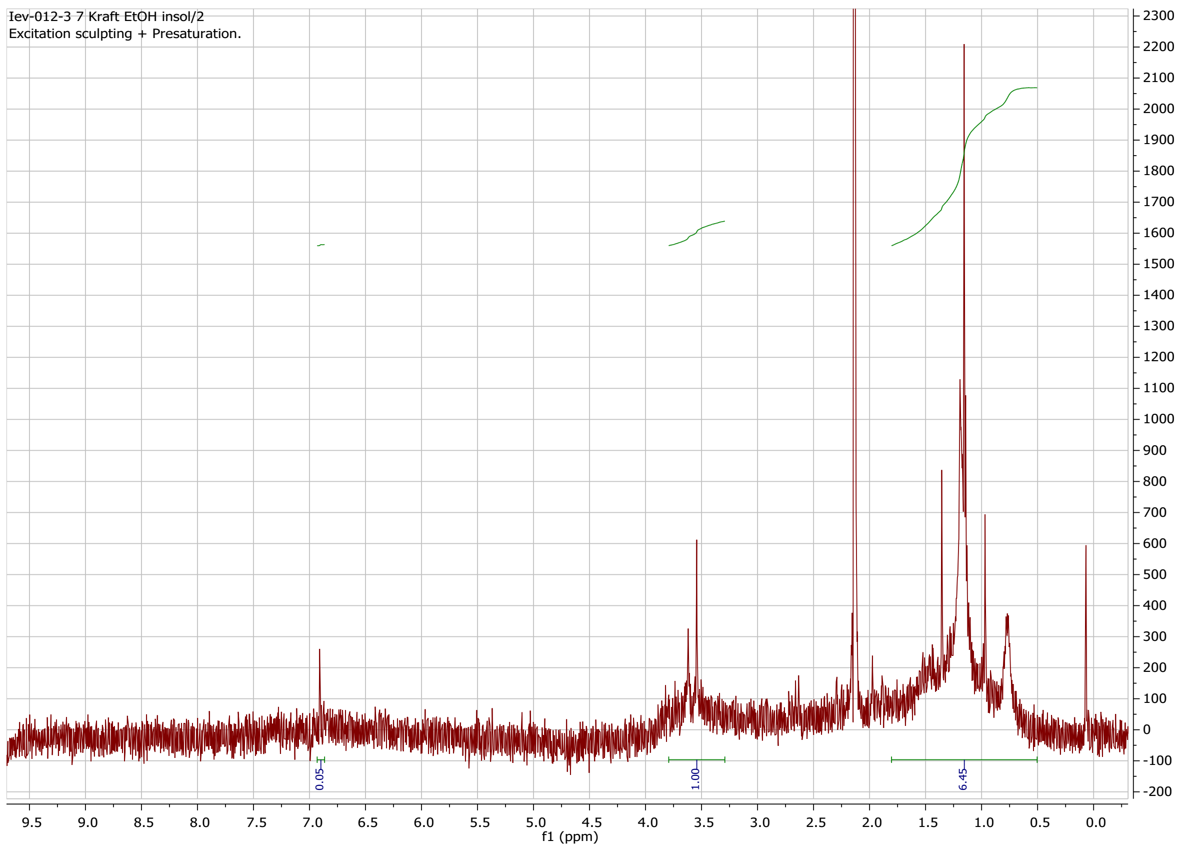

Figure S28. LNPs from kraft lignin, ethanol insoluble fraction.

Table S7. The integral intensities of some signals from the NMR of LNPs.

LNPs from	Relative integral intensity at **3.80-3.30 ppm** (-OCH_3 from S- and G- units)	Relative integral intensity at **1.80-0.5 ppm** (nonoxygenated aliphatic)	Relative integral intensity at (aromatics)
Ethanol soluble OS protected	1	61.52	dd 7.66-7.23 ppm 0.99+1.24 (coumaryl aldehyde and/or **Stilbene, β-5´, C_β**
Initial OS protected	1	1.15	-
Ethanol insoluble OS protected	1	17.56	-
Ethanol soluble OS Reference	1	6.9	

Initial<br>OS Reference	1	2.80	s<br>8.39-8.31ppm<br>0.20
Ethanol insoluble<br>OS Reference	1	2.33	
Ethanol soluble<br>Kraft	1	2.43	s 7.09-6.18 ppm<br>0.52<br>(**Stilbene, β-1´, $C_α$**<br>and/or<br>**Enol ether, $C_α$**
Initial<br>Kraft	1	0.02	
Ethnaol insoluble<br>Kraft	1	6.45	S 6.93-6.87 ppm<br>0.05<br>**Stilbene, β-1´, $C_α$**

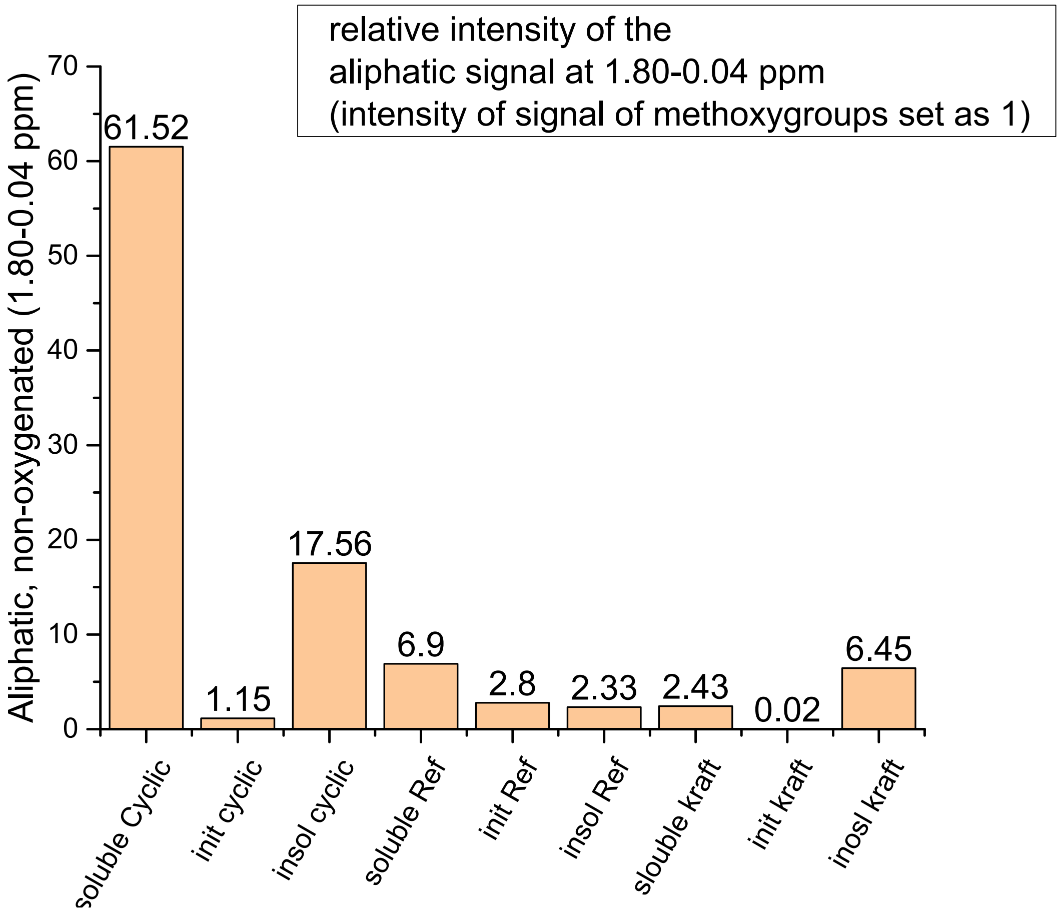

Figure S29. Relative intensity of the non-oxygenated aliphatic signal at 1.80-0.04 ppm (intensity of signal of methoxy groups set as 1). Please also look in Table S7.

6. Scheme of biorefinery processes

6.1. OS protected lignin

In relation to the organosolv lignins, cyclic extraction applies a biorefinery concept, where wood polymers are sequentially extracted, i.e., a stream of hemicellulose was extracted using subcritical water prior to the organosolv extraction of lignin. This biorefinery-based process aims to preserve the native lignin structure, where native-like lignin is extracted after the extraction of hemicelluloses at 160°C for 2 hours, using the principle of additive-free physical protection strategies during the extraction. The extraction was performed in fifteen cycles, each for five minutes, using an accelerated solvent extraction at a temperature of 160°C. This lignin is referred to as "protected lignin" where β-O-4' units are preserved.

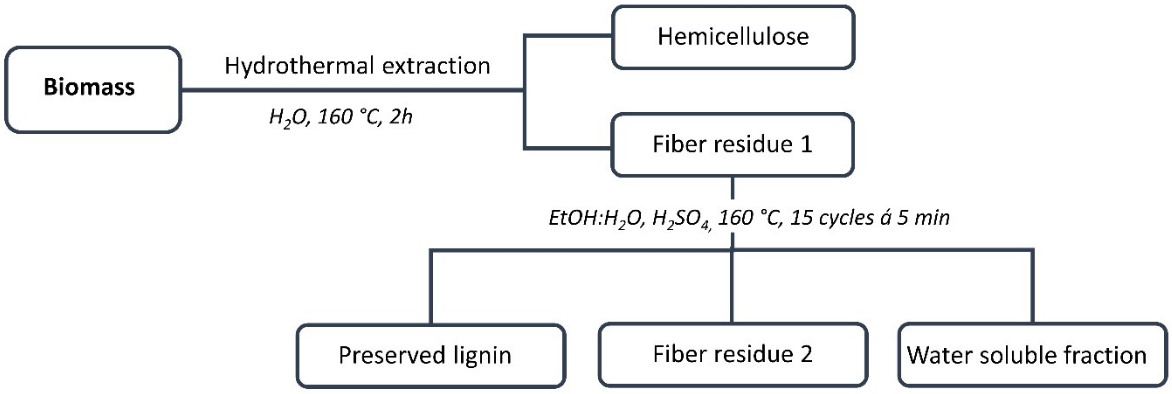

Figure S30. Scheme of the consolidated biorefinery process where lignin with preserved structure is extracted.

6.2. OS reference lignin

In the second biorefinery-based process, lignin was extracted based on an organosolv process subsequent to hemicellulose extraction, but in batch mode at 160°C for 2 hours. This lignin is referred to simply as "organosolv lignin". Overall, these modifications when compared to the classical one-step organosolv process without pre-extraction of hemicellulose, improved the lignin purity.

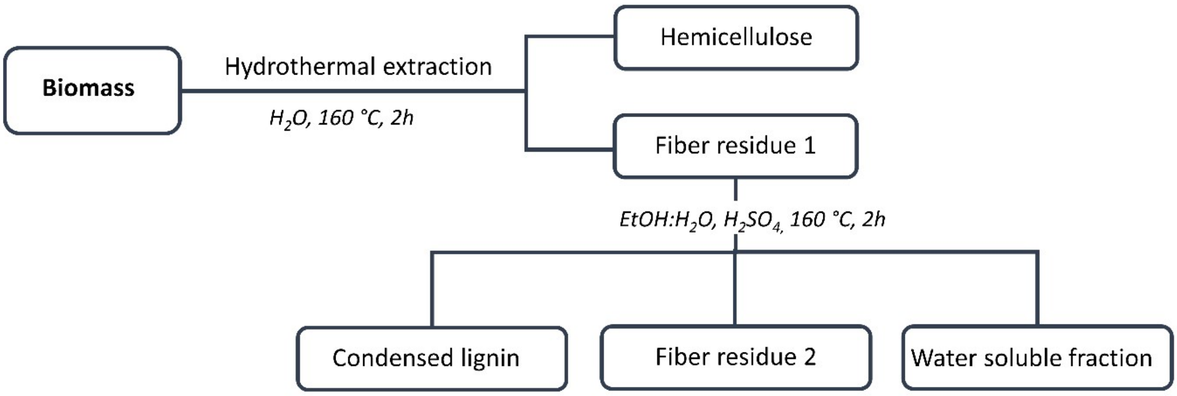

Figure S31. Scheme of the reference consolidated biorefinery process where a batch lignin is extracted.

7. 31P NMR spectra of ethanol fractions

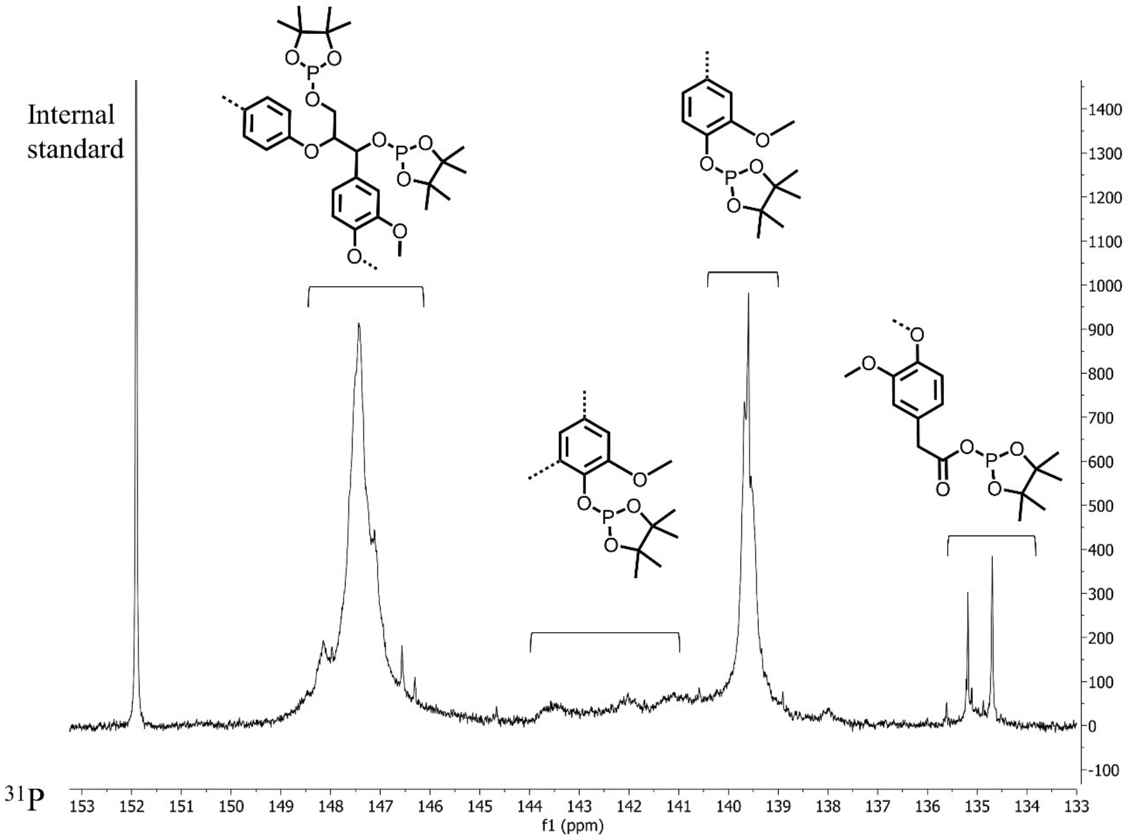

Figure S32. ^{31}P NMR spectra of the ethanol soluble part of the fractionated OS protected lignin.

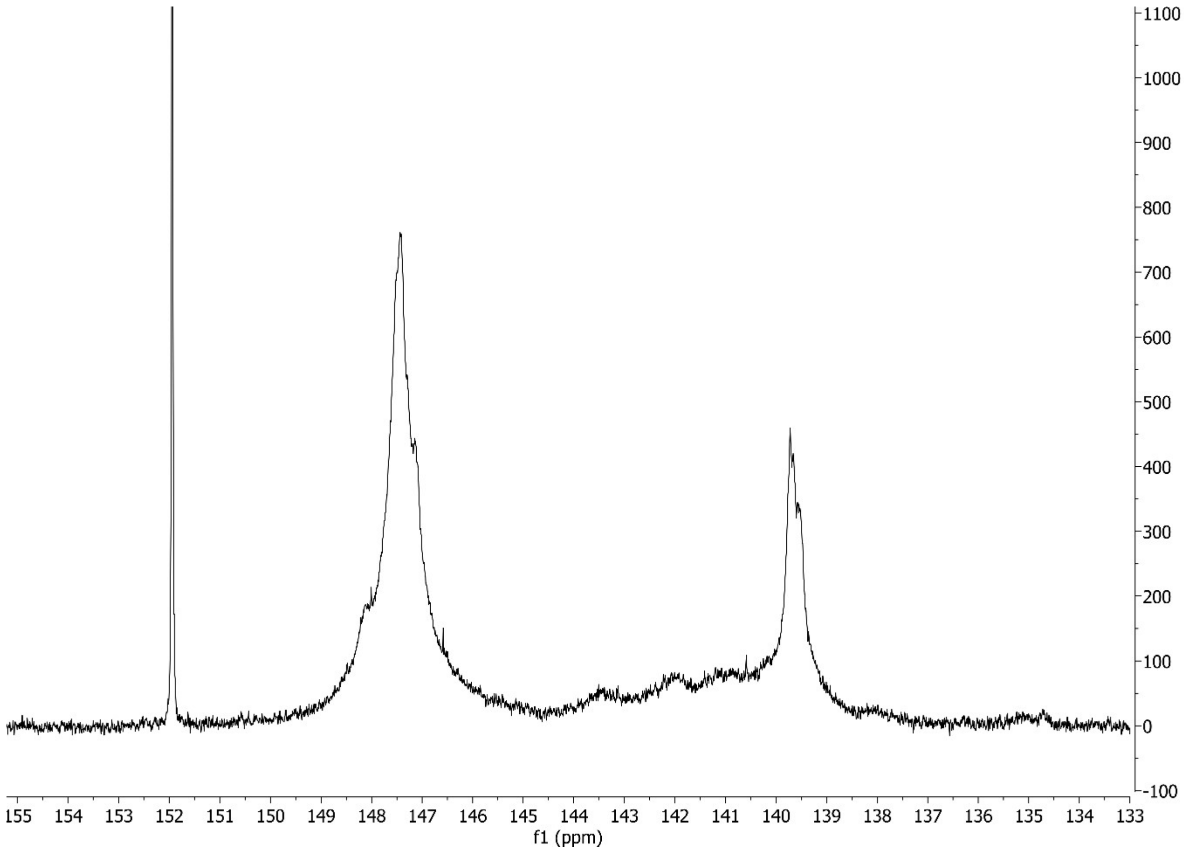

Figure S33. ^{31}P NMR spectra of the ethanol insoluble part of the fractionated OS protected lignin.

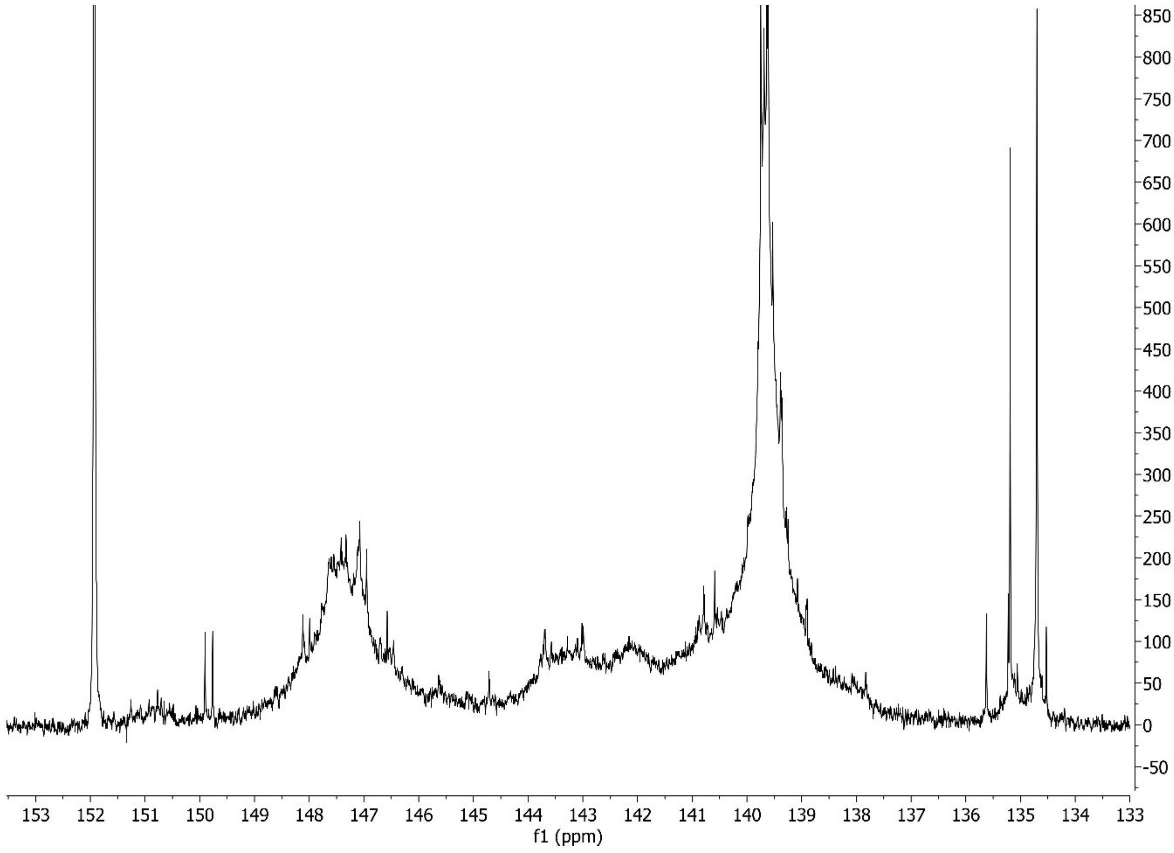

Figure S34. ^{31}P NMR spectra of the ethanol soluble part of the fractionated OS reference lignin

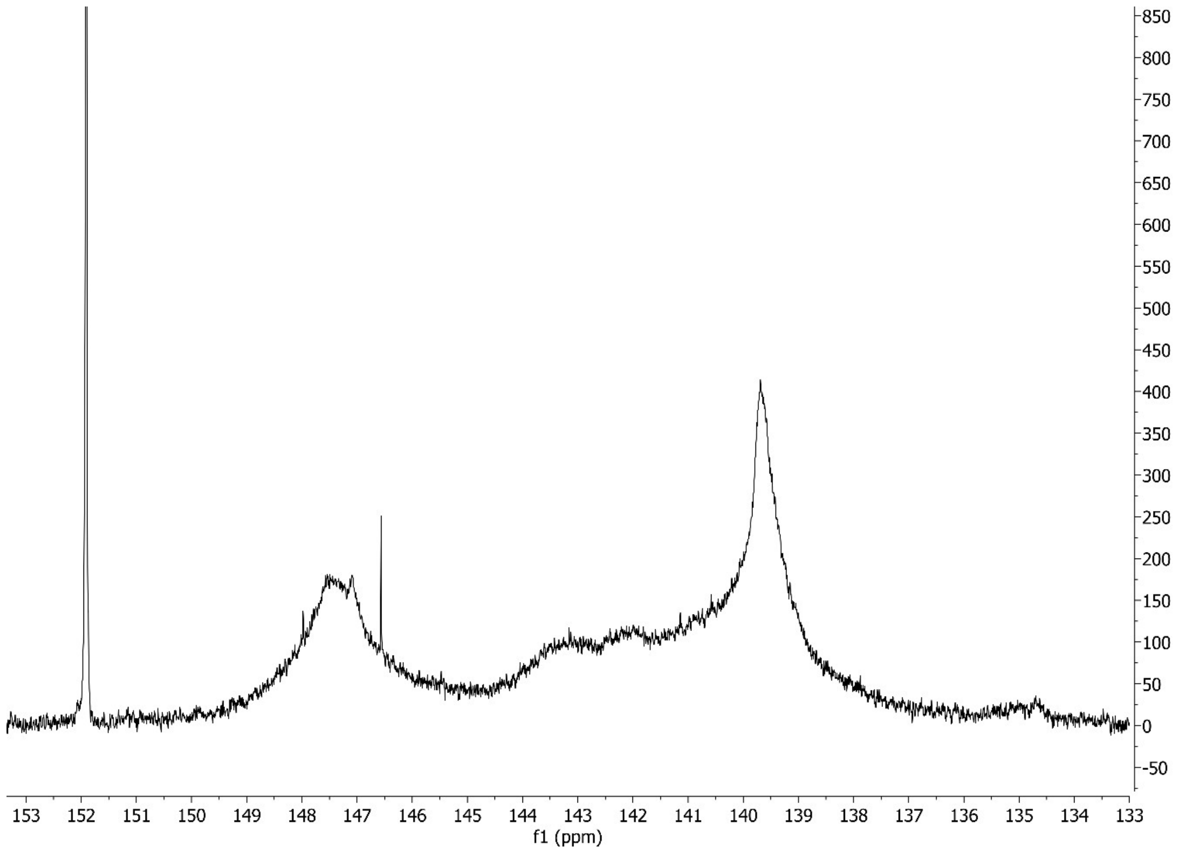

Figure S35. ^{31}P NMR spectra of the ethanol insoluble part of the fractionated OS reference lignin.

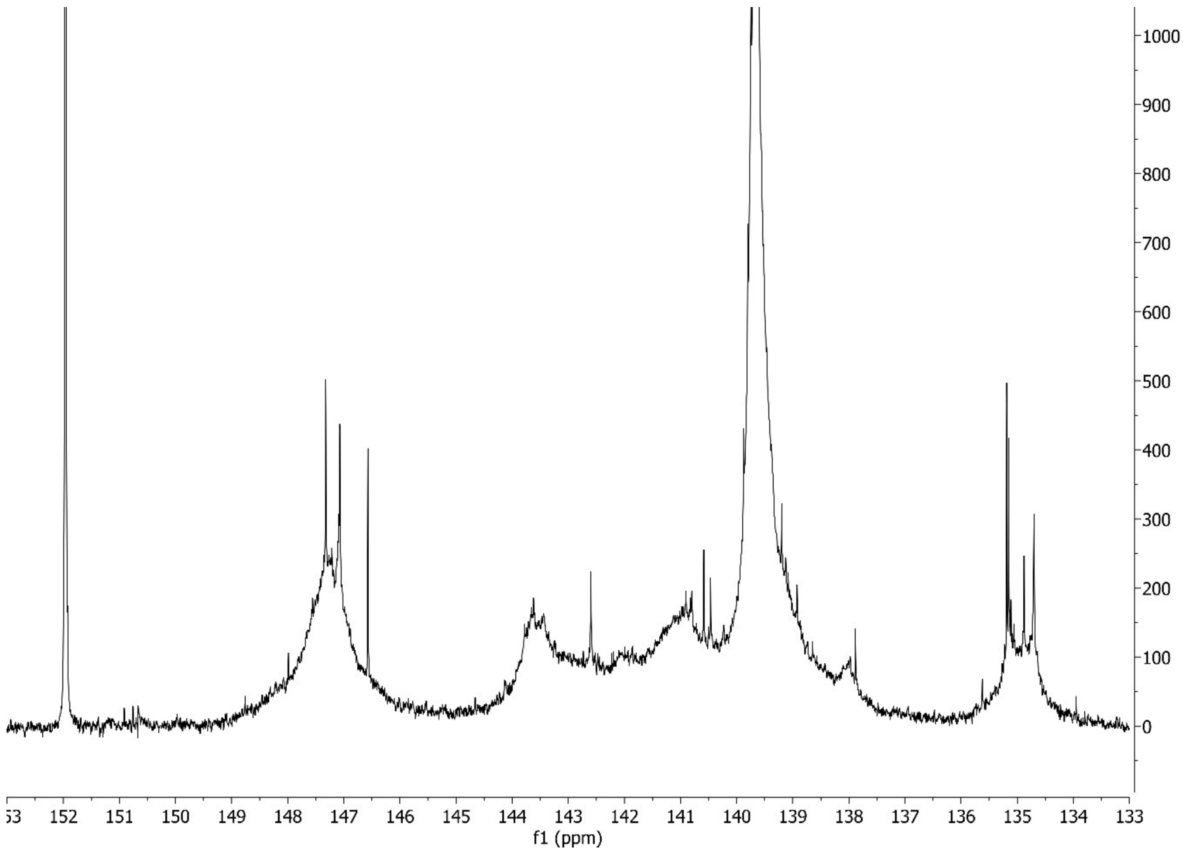

Figure S36. ^{31}P NMR spectra of the ethanol soluble part of the fractionated kraft lignin.

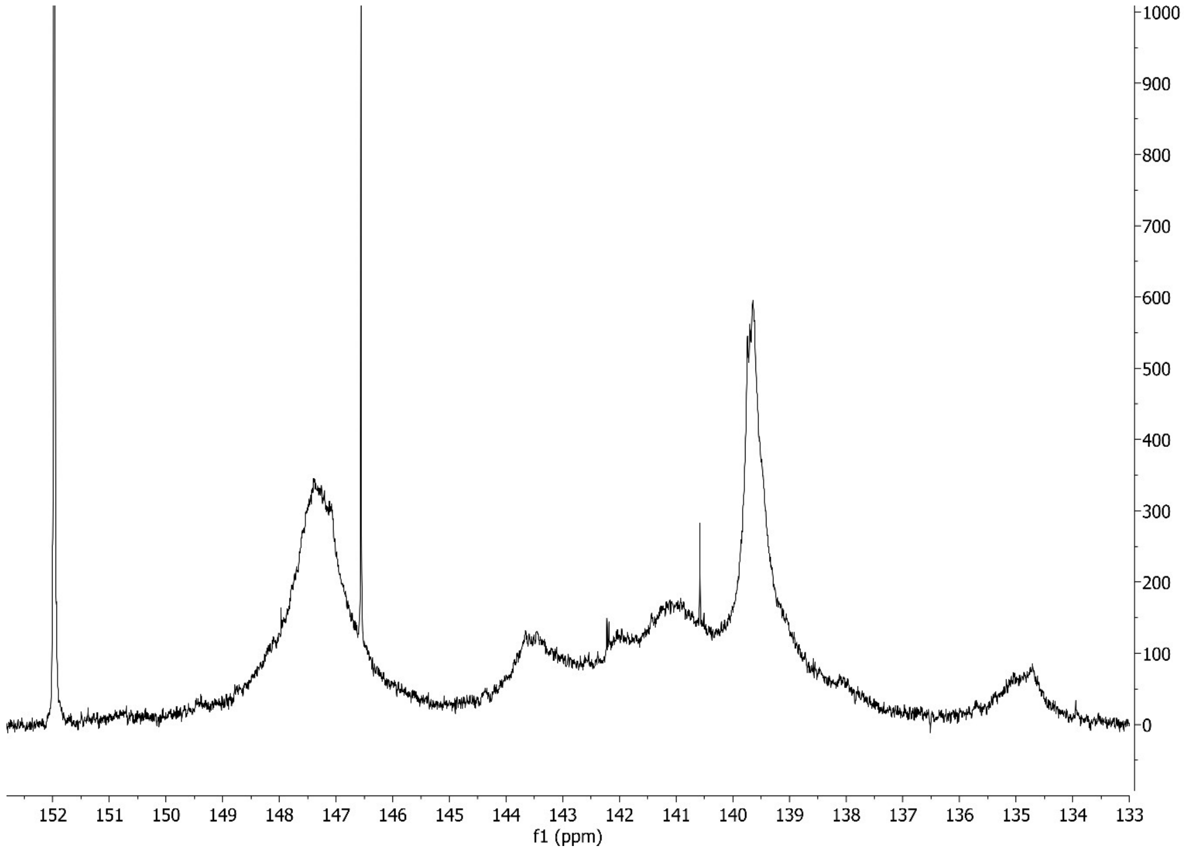

Figure S37. ^{31}P NMR spectra of the ethanol insoluble part of the fractionated kraft lignin.

8. Extraction and ethanol fractionation yield

Table S8. The absolute dry mass% of the fractions after ethanol fractionation.

Lignin	Extracted lignin yield [mass%][1]	EtOH soluble [mass%]	EtOH insoluble [mass%]
OS protected	54	45.8	53.4
OS reference	48	45.4	53.3
Kraft	-	42.7	56.7

[1]Assuming 27% lignin in spruce wood.

9. Lignin structures

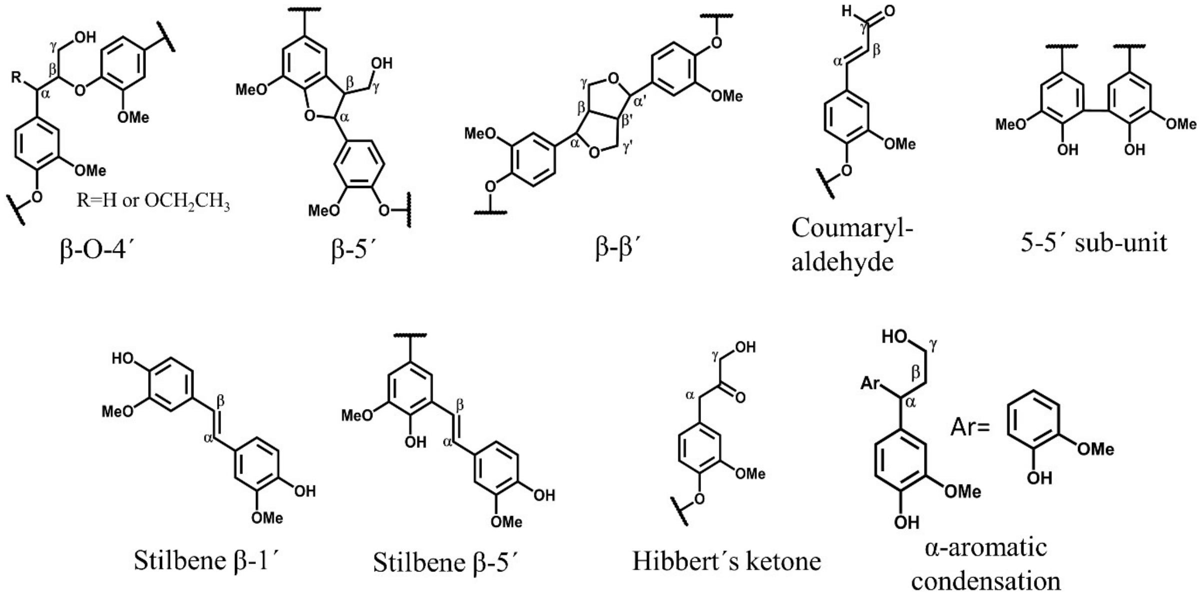

Figure S38. Identified structures in the lignin fractions.

10. DFT: Molecular volume

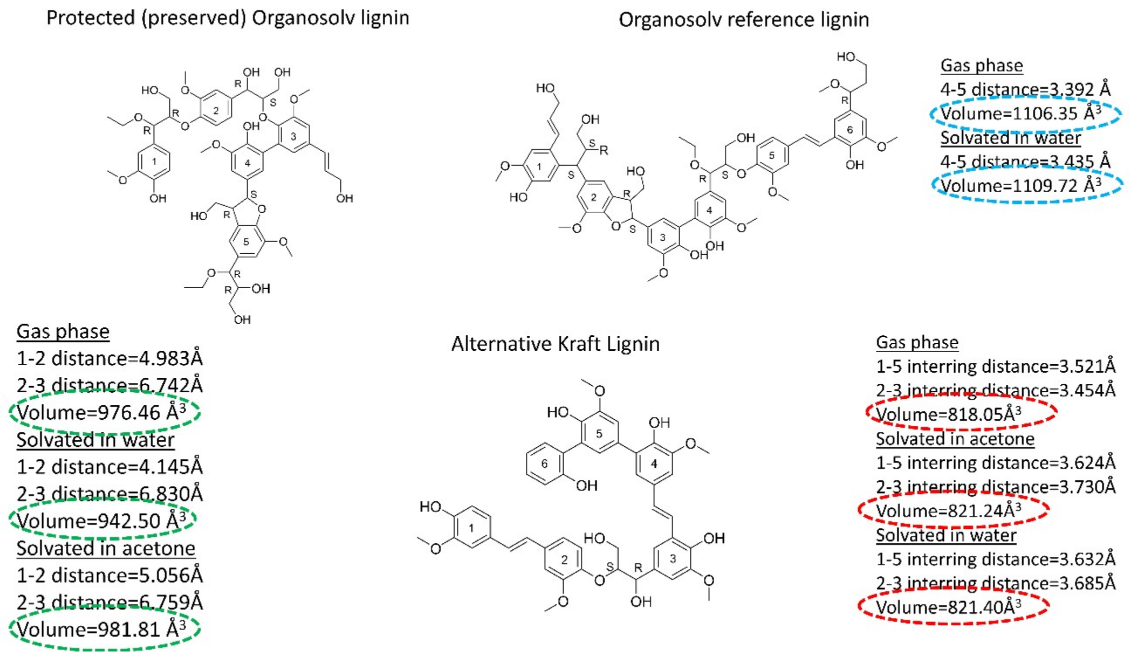

Figure S39. Molecular volume in different systems.
